# Combining short and long read sequencing to characterize antimicrobial resistance genes on plasmids applied to an unauthorized genetically modified *Bacillus*

**DOI:** 10.1038/s41598-020-61158-0

**Published:** 2020-03-09

**Authors:** Bas Berbers, Assia Saltykova, Cristina Garcia-Graells, Patrick Philipp, Fabrice Arella, Kathleen Marchal, Raf Winand, Kevin Vanneste, Nancy H. C. Roosens, Sigrid C. J. De Keersmaecker

**Affiliations:** 1 Transversal activities in Applied Genomics, Sciensano, Brussels, Belgium; 2 Department of Information Technology, IDLab, Ghent University, IMEC, Ghent, Belgium; 3 Foodborne Pathogens, Sciensano, Brussels, Belgium; 4 Service Commun des Laboratoires, Illkirch-Graffenstaden, France; 5 Department of Plant Biotechnology and Bioinformatics, Ghent University, Ghent, Belgium

**Keywords:** Mobile elements, Next-generation sequencing, Antimicrobial resistance, Bacterial genomics, Policy and public health in microbiology

## Abstract

Antimicrobial resistance (AMR) is a major public health threat. Plasmids are able to transfer AMR genes among bacterial isolates. Whole genome sequencing (WGS) is a powerful tool to monitor AMR determinants. However, plasmids are difficult to reconstruct from WGS data. This study aimed to improve the characterization, including the localization of AMR genes using short and long read WGS strategies. We used a genetically modified (GM) *Bacillus subtilis* isolated as unexpected contamination in a feed additive, and therefore considered unauthorized (RASFF 2014.1249), as a case study. In GM organisms, AMR genes are used as selection markers. Because of the concern of spread of these AMR genes when present on mobile genetic elements, it is crucial to characterize their location. Our approach resulted in an assembly of one chromosome and one plasmid, each with several AMR determinants of which five are against critically important antibiotics. Interestingly, we found several plasmids, containing AMR genes, integrated in the chromosome in a repetitive region of at least 53 kb. Our findings would have been impossible using short reads only. We illustrated the added value of long read sequencing in addressing the challenges of plasmid reconstruction within the context of evaluating the risk of AMR spread.

## Introduction

Antimicrobial resistance (AMR) genes are naturally present in bacteria, where they function as a defense mechanism. However, the overuse of antibiotics in humans and animals over several decades has led to a rapid rise in the prevalence of AMR genes and the emergence of new AMR mechanisms. The monitoring of AMR is of the utmost importance to have an overview of the circulating resistance genes to implement policies to reduce AMR^1,2^.

The gold standards for AMR detection are phenotypic susceptibility tests and genotypic (q)PCRs. These methods lack the flexibility to continuously search for new mutations/genes and can be very time-consuming. The revolution in DNA-sequencing technologies, i.e. the so-called whole genome sequencing (WGS) technologies combined with specific databases^3^, offers a solution as an efficient, high-throughput analysis method for the characterization of AMR genes.

AMR genes can be present on the chromosome or on mobile elements, such as plasmids. Plasmids facilitate the spread of AMR genes, due to their ability to transfer to other bacteria, which is even possible across the species barrier^4,5^. Despite the importance of plasmids for the monitoring of AMR genes, it has been shown that they are difficult to reconstruct using second generation (i.e. short read) WGS data due to many repeats occurring in the plasmid and that are sometimes even shared with the chromosomal DNA^6,7^. Therefore, it is difficult to determine the exact location of AMR genes when using short read WGS-based approaches. Nevertheless, this information is needed for a full risk assessment of AMR transmission. The use of long read sequencing such as offered by PacBio and Oxford Nanopore Technology (ONT) would be of added value in this context, as the repetitive regions can be spanned by the long reads generated by this technology^8^. However, the rather high error rate of ONT^9^ would benefit from the combination of the long reads with the accuracy of short read sequencing^10^. This kind of hybrid assembly approach was shown previously to be effective in reconstructing accurate, contiguous genomes, including plasmids containing AMR genes in (pathogenic) wild-type bacteria^10–13^.

Although not yet reported to be thoroughly studied in this field, the issue of AMR gene location is especially important in the context of genetically modified microorganisms (GMMs). AMR genes are often used as selection markers for the integration of their newly introduced target genes, needed to produce a specific food or feed additive, such as vitamins, through fermentation or to make them more safe by suppressing the synthesis of toxins^14–16^. Due to technological advances in genetic engineering, it is possible to delete regions from the chromosome or insert foreign DNA. The insertion of genes is done to disrupt the endogenous genes or to more stably express foreign genes. In some cases this is done by plasmids where regions of the plasmid or the entire plasmid are integrated in the chromosome by homologous recombination. However, sometimes it is more beneficial to design plasmids in a way that they remain as an extra-chromosomal element in the bacterial cell without integration of their sequences. So, both scenarios are possible, i.e. plasmid DNA extra-chromosomally present or integrated in the chromosome of the GMM.

There exists a complex regulatory framework for the placement of GMMs or derived products and substances in the European market. Producer companies of GMMs used in the food chain or as producer organisms for substances of interest should submit an application to the European Food Safety Authority (EFSA) for safety evaluation, covering the characterization of the microorganisms used as producer organism, to get authorization for the marketing of their products. Recently, in the guidance of EFSA on the characterization of GMMs^14^, there is a newly introduced section that focuses on the fact that GMMs should not contribute to the pool of resistance against antimicrobials that are clinically relevant for their use in humans and animals. To protect consumers against potential adverse effects, viable GMMs should be absent in the microbial fermentation products commercialized on the European (EU) market (EC/1831/2003, EC/1332/2008, EC/1333/2008, EC/1334/2008)^17–20^. Moreover, if a contamination of a GMM is present in e.g. vitamins commercialized on the EU market, they are consequently falling under EU regulations related to the commercialization of genetically modified food and feed (2001/18/EC; 1829/2003/EC; 1830/18/2003)^21–23^. As there is no dossier submitted to EU for this use in the context of these regulations, this GMM is per se unauthorized and zero tolerance, including for its associated recombinant DNA, must be applied. Besides this regulatory aspect, AMR gene transmission also poses health and environmental concerns. The presence of full AMR genes in food/feed additives cannot only lead to direct transmission along the food chain to pathogens, but AMR genes can also spread to environmental reservoirs^24–26^.

Therefore, enforcement laboratories are starting to be involved in the official control of these GMM-derived products, to detect unexpected contaminations which make the GMM unauthorized. However, this detection is not an easy task as the dossier filed to EFSA containing the sequence information is confidential. Therefore, the information to develop the necessary qPCR methods targeting the specific GM events is not readily available^27^. Also because, unlike for plant GMOs, the companies do not have to provide any information to trace this GMO in the food and feed chains. Therefore, whole genome sequencing (WGS) becomes one of the major tools available to enforcement laboratories to characterize a GMM.

In 2014, a living GM *Bacillus subtilis* was detected in a feed additive that was capable of overproducing riboflavin (vitamin B2). Therefore, this GMM was unauthorized and consequently, a European Rapid Alert System for Food and Feed (RASFF) notification was created to alert other European countries (code 2014.1249). This GMM was imported from China and was distributed to up to 11 European countries. In 2015, the GM *Bacillus* strain (isolate 2014-3557) was isolated and WGS was applied (Illumina HiSeq2500, 2 × 125 bp) which was used to develop a qPCR method to quickly detect this GMM in other feed additives^27,28^. In an attempt to further characterize the strain, the genome of the same isolate was sequenced in 2017 (with Illumina MiSeq2 × 150 bp, HiSeq2 × 50 bp and GS junior System 400–600 bp) by two German enforcement laboratories^29^. These reads were used for the assembly of 4 GM plasmids (called pGMsub01–04), which contained the *aadD* (aminoglycoside resistance), *blaTEM-116* (beta-lactam resistance), *tet(L)* (tetracycline resistance) and *erm(B)* (erythromycin resistance) genes, although pGMsub03 and pGMsub04 were not detected by one of the two labs (‘LHL’). Moreover, in the chromosome, an insertion of a *cat* gene (chloramphenicol resistance) was detected. These AMR genes confer resistance to antibiotics that are determined by the WHO to be clinically relevant for human use^30^. Furthermore, qPCRs were developed for the detection of the event-specific integration of the *cat* gene in the chromosome and for the specific detection of the GM plasmids, to be used by the enforcement laboratories. In the supplementary information of the publication^29^, several claims by the Chinese producer of the GMM were mentioned. However, the claim that there was an integration of five pUC19 plasmids in the chromosome did not correspond with the assemblies presented by the authors^29^.

Because of these inconsistencies, we hypothesized that as only short read sequencing technologies were used in the assembly of the GM plasmids, the reads might not be able to completely cover the repetitive regions. Therefore, we used the aforementioned unauthorized GMM 2014-3557 as a case study to deliver a proof of concept for a WGS strategy to fully characterize all AMR genes and their exact location. To bridge the gaps of repetitive regions, we used long read sequencing technologies (ONT and PacBio) and a combination of short (MiSeq) and long reads (hybrid assemblies). This approach of hybrid assembly has not yet been reported to be applied on GMMs. Furthermore, we verified the assembly using PCR and qPCR and we determined its phenotype with antibiotic susceptibility and riboflavin dosage tests in comparison to the wild-type *B. subtilis*.

## Results

### Characterization of GM *B. subtilis* 2014-3557 at the genotypic level

In an attempt to better span repetitive regions, we used paired-end MiSeq and ONT MinION reads of the GM *B. subtilis* DNA to make a *de novo* hybrid assembly. The total assembly consisted of 2 circular contigs with a total size of 4,279,307 base pairs and a GC% of 43.53. The chromosome (contig 1) is 4,240,660 bp and the plasmid (pGMrib, contig 2) 38,647 bp. Both contigs were determined to be circular (supplementary information, Tables S3 and S4).

In the nucleotide database of NCBI, contig 1 (chromosome) was most similar to the *Bacillus subtilis* 168 genome (accession number: NZ_CP010052.1) while contig 2 (pGMrib) was most similar to the previously reported pGMsub04 (accession number: LT622643.1)^29^. Moreover, 99.9% of the *de novo* assembly aligned to the published scaffolds from Barbau-Piednoir *et al*.^28^.

**Figure 1:**
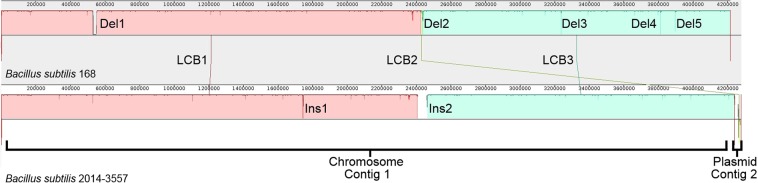
Mauve progressive comparison between wild-type *B. subtilis* 168 (NZ_CP010052.1) and the *de novo* assembly of the GM *B. subtilis* 2014-3557. Locally Collinear Blocks (LCBs) are shown in colour and positions where no colour is shown means that there is a deletion (Del) or insertion (Ins) in the GM *B. subtilis* 2014-3557 compared to the wild-type *B. subtilis* 168. LCB2 is present in pGMrib of GM *B. subtilis* 2014-3557 in the opposite orientation. The vertical red lines indicate where a contig begins or ends.

When comparing the chromosomes of the wild-type *B. subtilis* 168 (NZ_CP010052.1) and the GM 2014-3557 (contig 1) into more detail (Fig. 1), 2 insertions and 5 deletions were found in the GM 2014-3557 genome, in addition to 520 SNPs (supplementary information, Table S6).

**Figure 2:**
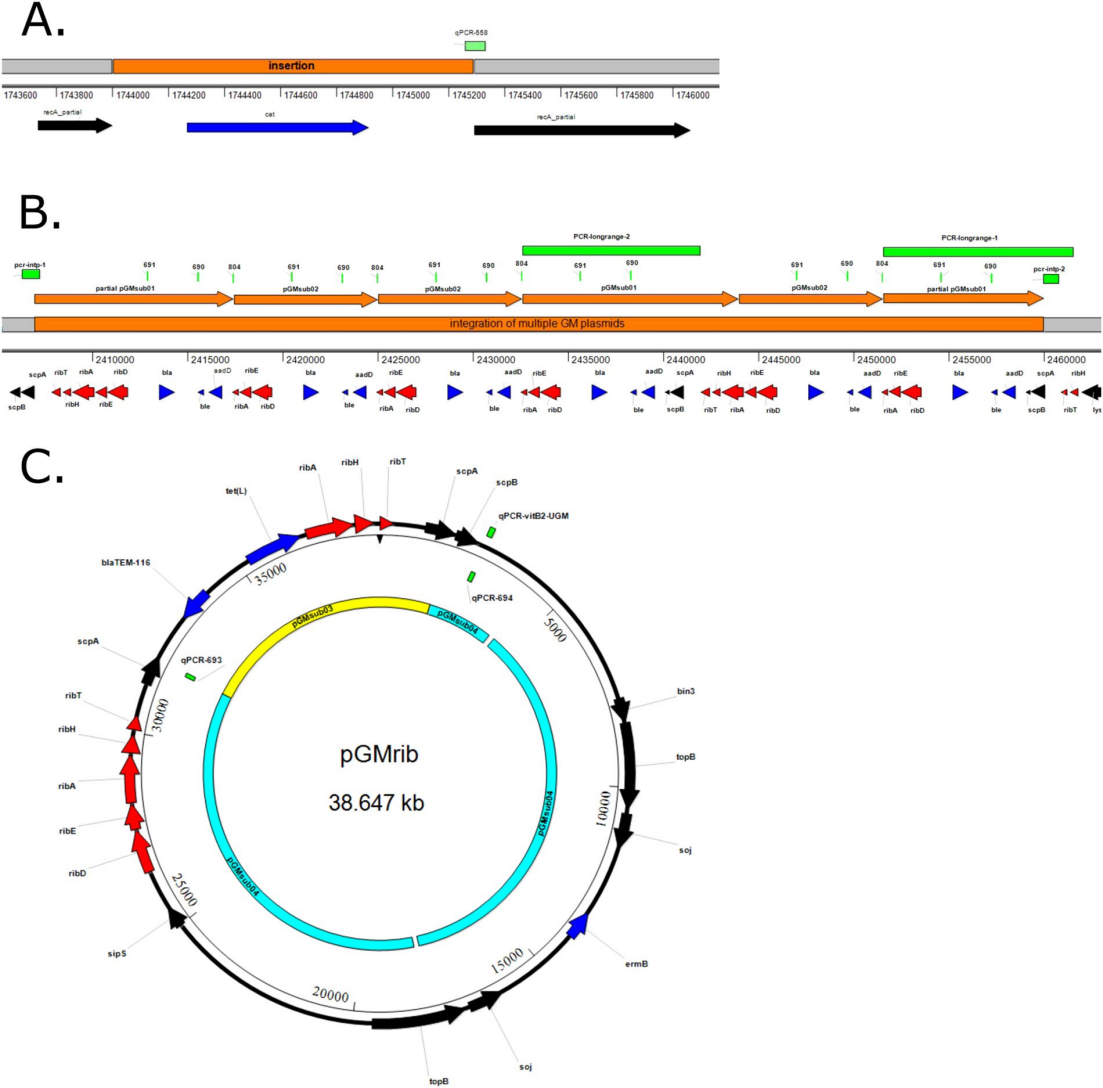
Genotypic characterization of the GM *B. subtilis* 2014-3557. (**A**) The insertion of the complete chloramphenicol resistance gene (*cat*) (originating from the plasmid pC194^31^) interrupting the sequence of the *recA* gene and the qPCR-558 assay^29^ that was developed to detect the junction of the *cat* insertion. (**B**. The insertion of multiple GM plasmids (pGMsub01 and pGMsub02) inside the chromosome and the PCR assays that were developed in this study to confirm the integration (PCR-longrange-1, PCR-longrange-2, PCR-intp-1 and PCR-intp-2). The integration consists of sequences from pUC19, pUB110, partial and complete *rib* operons from *B. amyloliquefaciens*. qPCR primers developed previously^29^ to detect pGMsub01 and pGMsub02 could bind in this region multiple times (assay 690, 691 and 804). (**C**). The circular GM plasmid pGMrib, which consists of sequences originating from pLS1, pSM19035, pUC19 and partial and complete *rib* operons from *B. subtilis* 168. In yellow and light blue a MauveProgressive comparison of pGMrib to pGMsub03 and pGMsub04^29^. The qPCR assays developed for the specific detection of the plasmid (qPCR-693, qPCR-694^29^ and qPCR-vitB2-UGM^27^) are indicated. Color codes in all panels: Genes in red are associated with riboflavin production, genes in blue are associated with antibiotic resistance, genes in black are all other genes, in light green are (q)PCR amplicons^27,29^ and in orange are the plasmid sequences inserted in the chromosome.

At position 1,744,001 (Fig. 2A), there is a 1,288 bp insertion, i.e. the entire *cat* gene originating from the plasmid pC194^31^ is inserted in the *recA* gene. The *recA* gene is involved in homologous recombination and DNA repair. At position 2,406,920 a 53 kb insertion occurs (Fig. 2B) within the *scpA* gene. Within this integration, several occurrences of the full *ribDEAHT* and partial *ribDEA* operons were detected. These operons differ from the natural occurring *rib* operon in *B. subtilis* and they showed the highest similarity to the *rib* operon of *Bacillus amyloliquefaciens*. In addition, multiple copies of beta-lactamase and kanamycin resistance genes were found in the 53 kb insertion. Lastly, a bleomycin resistance gene was found.

Upon further inspection of the repetitive insertion at position 2,406,920–2,459,995 we found by using BLAST that it contained 3 repetitions of pGMsub02 (7,581 bp), once pGMsub01 (11,378 bp) and 2 partial sequences of the pGMsub01 plasmid (10,492 bp and 8,467 bp) (Fig. 2B). pGMsub01 and pGMsub02 were previously reported to be extra-chromosomal circular plasmids^29^, however, in the current *de novo* assembly, they were present integrated inside the chromosome (contig 1). As described previously^29^, pGMsub02 is the same as pGMsub01, with the only difference being a deletion of 3,797 bp in the *B. amyloliquefaciens rib* operon. Moreover, these GM plasmids contain sequences from the partial and complete *rib* operons of *B. amyloliquefaciens* (accession number: CP041693.1), pUC19 (accession number: M77789.2) and pUB110 (accession number: M19465.1).

At position 529,007 a region of 20,525 bp was deleted, removing the *int* (ICEBs1 integrase gene), *immA* (metallopeptidase gene), *immR* (HTH-type transcriptional regulator gene), *xis* (ICEBs1 excisionase gene), *nick* (putative DNA relaxase gene), *rapI* (response regulator aspartate phosphatase I gene) and several uncharacterized genes. The second deletion at position 2,461,930 is one of 8,460 bp, and occurs 1,935 bp after the above elaborated insertion of 53 kb. The sequence of *ribDEA* from the *ribDEAHT* operon is removed together with a riboflavin switch. Furthermore *sipS* (signal peptidase I S gene), *ypzD* (spore germination protein-like protein encoding gene) and *ppiB* (peptidyl-prolyl cis-trans isomerase B gene) were removed from this location. This deletion would make *B. subtilis* unable to produce riboflavin without additional genetic modifications. However, the full sequence of this deletion can be detected in the extra-chromosomal plasmid of 38 kb (contig 2). At position 3,261,217, there is a third deletion of 200 bp, interrupting a hypothetical protein. At position 3,837,414, a fourth deletion of 106 bp interrupts the sequence of the gene *rpoE* (DNA-directed RNA polymerase subunit delta). At position 3,921,625, a fifth deletion of 46 bp interrupts the sequence of *ywdH* (putative aldehyde dehydrogenase gene).

In Fig. 2C the annotation of the pGMrib (contig 2) is visualized. The plasmid contains 1 full *ribDEAHT* and 1 partial *ribAHT* operon from *B. subtilis*. Furthermore, it contains genes that were flanking the *rib* operon in *B. subtilis 168* chromosome, such as *scpA* and *scpB* (segregation and condensation genes). The plasmid contains *bla* (beta-lactamase resistance) from pUC19 plasmid sequences, *tet(L)* from pLS1 plasmid sequences (accession number: M29725.1) and *erm(B)* from pSM19035 plasmid sequences (accession number: AY357120.1). BLASTing contig 2 (plasmid) resulted in hits for both the complete pGMsub03 (8,544 bp) and pGMsub04 (29,760 bp), as depicted in Fig. 2C^29^. However, the part that matched with pGMsub04 was slightly longer and accounted for 30,104 bp.

The annotation of the contigs already revealed some AMR genes. To obtain the full picture of the AMR gene content, we performed a search on the *de novo* assembly using the ResFinder tool (Table 1). The chromosome (contig 1) contained 6 copies of *aadD*, 1 copy of *aadK*, 6 copies of *bla-TEM-116*, 1 copy of *mph(K)*, 1 copy of *cat(pC194)* and 1 copy of *tet(L)* gene, while the plasmid (contig 2) contains 1 copy of *erm(B)* and 1 copy of a different *tet(L)* gene. Only the *mph(K)*, *aadK* and the chromosomal *tet(L)* genes were naturally present on the *B. subtilis* 168 chromosome (NZ_CP010052.1). Therefore all other AMR genes were genetically inserted. Amongst the 520 SNPs detected (supplementary information, Table S6), we also found a SNP in the *rpsL* gene (A > G substitution), which is known to account for streptomycin resistance^32^.

**Table 1:** Antimicrobial resistance genes detected with ResFinder in wild-type *B. subtilis* 168 (NZ_CP010052.1) and GM *B. subtilis* 2014-3557.

Resistance gene	Identity	Alignment Length	Coverage	Contig	Position in contig	Accession no.
*B. subtilis* 168						
**Macrolide**						
*mph(K)*	100	921/921	100	chromosome	275,845..276,765	NC_000964
**Tetracycline**						
*tet(L)*	100	1377 / 1377	100	chromosome	4,187,694..4,189,070	X08034
**Aminoglycoside**						
*aadK*	100	855/855	100	chromosome	2,735,694..2,736,548	M26879
***B. subtilis*** **2014-3557**						
**Aminoglycoside**						
*aadD*	100	771/771	100	chromosome	2,457,772..2,458,542	M19465
*aadD*	100	771/771	100	chromosome	2,450,192..2,450,962	M19465
*aadD*	100	771/771	100	chromosome	2,438,814..2,439,584	M19465
*aadD*	100	771/771	100	chromosome	2,431,234..2,432,004	M19465
*aadD*	100	771/771	100	chromosome	2,423,654..2,424,424	M19465
*aadD*	100	771/771	100	chromosome	2,416,074..2,416,844	M19465
*aadK*	100	855/855	100	chromosome	2,760,674..2,761,528	M26879
**Beta-lactam**						
*blaTEM-116*	100	861/861	100	chromosome	2,413,471..2,414,331	AY425988
*blaTEM-116*	100	861/861	100	chromosome	2,421,051..2,421,911	AY425988
*blaTEM-116*	100	861/861	100	chromosome	2,428,631..2,429,491	AY425988
*blaTEM-116*	100	861/861	100	chromosome	2,436,211..2,437,071	AY425988
*blaTEM-116*	100	861/861	100	chromosome	2,447,589..2,448,449	AY425988
*blaTEM-116*	100	861/861	100	chromosome	2,455,169..2,456,029	AY425988
*blaTEM-116*	100	861/861	100	plasmid	33,086..33,946	AY425988
**Macrolides**						
*mph(K)*	100	921/921	100	chromosome	275,437..276,357	NC_000964
*erm(B)*	100	738/738	100	plasmid	13,276..14,013	U86375
**Phenicol**						
*cat(pC194*)	100	651/651	100	chromosome	1,744,265..1,744,915	NC_002013
**Tetracycline**						
*tet(L)*	100	1377/1377	100	chromosome	4,212,326..4,213,702	X08034
*tet(L)*	100	1377/1377	100	plasmid	35,241..36,617	M29725

With PlasmidFinder, the replication origin *repS* (X64695) was detected at position 5,770–7,260 of contig 2. No plasmid replicon was found in contig 1.

### Confirmation of the genetic modifications in GM *B. subtilis* 2014-3557

As the *de novo* hybrid assembly of the GM *B. subtilis* 2014-3557 was different compared to the previously reported characterization^29^, we performed additional analyses to confirm the integration of the plasmids in the chromosome.

#### Evidence from sequencing reads

First, a PacBio (sequel) run on the wild-type *B. subtilis* 168 and the GM *B. subtilis* 2014-3557 was performed, in an attempt to obtain longer, more accurate reads. However, after comparison to the *de novo* hybrid assembly (from MiSeq and MinION reads) it was surprisingly found that the latter was more accurate and contiguous (Table S3). This can likely be attributed to the higher average size of the MinION reads (7,731 bp vs. 4,347 bp). Nevertheless, the PacBio reads were useful for further validation of the *de novo* hybrid assembly. Indeed, the MiSeq, MinION and PacBio reads were mapped to the hybrid assembly to check for coverage and irregularities (insert size, proper pairing of paired end reads and clipping of long reads) in the assembly. The whole assembly was completely covered by the 3 types of reads. Up to 99.9% of the MiSeq and MinION reads and 98.97% of the PacBio reads mapped to the *de novo* assembly. With long reads (supplementary information, Figures S1 and S2), the entire GM plasmid was covered by uniquely mapped reads. Reads from all three technologies could be uniquely mapped to the insertion sites of the 53 kb repetitive region. However, the average mapping quality in the 53 kb repetitive region was higher for the MinION reads compared to both MiSeq and PacBio reads (supplementary information, Figures S1 and S2). The average depth of uniquely mapped MinION reads in the 53 kb repetitive region (2,406,920–2,459,995) was 74 × (SD 58). As there was not a single read that could span the entire repetitive region, it is possible that this region is longer than 53 kb. However, long MinION and PacBio reads (>12 kbp) were found to cover unique regions in the 53 kb insertion (supplementary information, Table S5).

#### Evidence from qPCR for the integration of GM plasmids in the chromosome

Next, we investigated the integration of the previously reported plasmids^29^ in the GM *B. subtilis* 2014-3557 chromosome using qPCR assays that were previously developed by Paracchini *et al*.^29^. The Cq values of two specific qPCR reactions were compared (Table 2) to determine the difference in copy number, i.e. assay 558 (Fig. 2A)^29^ detecting the integration of the *cat* gene in the *Bacillus* chromosome and assay 804 (Fig. 2B)^29^ detecting the GM plasmid pGMsub02 that is integrated multiple times in the chromosome. Based on an *in silico* analysis, if the pGMsub01–02 plasmids would be integrated in the chromosome, then assay 804 should produce 4 times more amplicons than assay 558. We obtained for the GM *B. subtilis* 2014-3557 a Cq difference of 1.89 (SD 0.06) between assay 558 and 804 (Table 2), which indicates a 4-fold copy number difference, assuming that the qPCR efficiency of both assays is 100%. The DNA of the wild-type *B. subtilis* 168 yielded no detectable amplicon, as expected (Table S2).

**Table 2:** Cq difference between assay 558 and 804 on the DNA from *B. subtilis* 2014-3557 to investigate GM plasmid integration.

qPCR assay	DNA of isolate	amount of input DNA (ng)	Average c_q_	Δc_q_	Calculated copy number difference*	Expected copy number difference
558	GM *B. subtilis* 2014-3557	5	18.38 (SD 0.02)	1.89 (SD 0.06)	3.71 (SD 0.15)	4
804	GM *B. subtilis* 2014-3557	5	16.49 (SD 0.06)			

The primers of assay 558 target the *cat* gene integration and the primers of assay 804 target GM plasmid pGMsub02 that according to our de novo hybrid assembly is integrated in the chromosome.

*Assuming a 100% efficiency of the qPCR assay.

#### Evidence from PCR for the integration of GM plasmids in the chromosome

Finally, the integration of the plasmid sequences (pGMsub01 and pGMsub02) in the chromosome, was confirmed using 2 conventional PCRs (PCR-intp-1 and PCR-intp-2, Fig. 2B) targeting the insertion sites. Additionally, long-range PCRs (PCR-longrange-1 and PCR-longrange2, 9 and 10 kb products) that cover regions within the repetitive 53 kb insertion (positions 2,432,562–2,441,949 and 2,451,520–2,461,558) were developed (Fig. 2B). The 4 PCR assays tested positive in the GM *B. subtilis* 2014-3557 with the expected amplicon size and negative in the *B. subtilis* 168 (Table 3). Furthermore, a PCR assay (PCR-longrange-3) was developed spanning both the 5′ and 3′ end of the 53 kb integration. This PCR assay only produced an amplicon in the wild-type *B. subtilis* 168, because the region was too long to be amplified by PCR in the GM *B. subtilis* (Table 3).

**Table 3:** PCRs tested on the wild-type *B. subtilis* 168 and GM *B. subtilis* 2014-3557, their expected and obtained amplicon size.

target	name	expected size without genetic modifications (bp)	expected size with genetic modifications (bp)	detected size in *B. subtilis* 168 (bp)	detected size in *B. subtili*s 2014-3557 (bp)
Integration of GM plasmids 5′ side	PCR-intp-1	no amplicon	988	no amplicon	955
Integration of GM plasmids 3′ side	PCR-intp-2	no amplicon	868	no amplicon	840
unique region in repetitive integrated plasmids	PCR-longrange-1	no amplicon	9,338	no amplicon	11,801
unique region in repetitive integrated plasmids	PCR-longrange-2	no amplicon	10,039	no amplicon	12,399
No chromosomal integration of GM plasmids	PCR-longrange-3	2,316	>53 kb, not detectable with PCR	2,222	406*

The size of the amplicon was determined using the genomic screentapes of the Tapestation. The detected sizes have an accuracy of +/−15%.

*Confirmed with Sanger sequencing to be an aspecific product.

No additional PCRs for the confirmation of the pGMrib were developed as the coverage of the long reads was high and all long reads mapped uniquely in this contig (supplementary information, Figures S1 and S2). This included multiple MinION and PacBio reads of >9 kb that covered the connection between pGMsub03 and pGMsub04, demonstrating that these were indeed part of one plasmid.

### Phenotypic characterization of GM *B. subtilis* 2014-3557

As previously reported^27–29^, a phenotypic difference between wild-type and GM *B. subtilis* was seen during the culturing step. *B. subtilis* 2014-3557 displayed a distinctive yellow colour, whereas the wild-type *B. subtilis* did not show any colour (supplementary information, Fig. S3). As the GM *B. subtilis* was found to contain a full *rib* operon (containing essential genes for the production of riboflavin) and riboflavin is known to have a yellow colour, this observation indicated an overproduction of riboflavin by the GM *B. subtilis*. This was confirmed by the dosage tests of the riboflavin produced by the GM *B. subtilis*, compared to a wild-type *B. subtilis* (Table 4).

**Table 4:** Average amount of riboflavin produced by wild-type *B. subtilis* and GM *B. subtilis* 2014-3557.

	Average dosage riboflavin (mg/100 g)
WT *B. subtilis*	0.0188
GM *B. subtilis* 2014-3557	1.2124

The blanc yielded 0.0189 mg/100 g of riboflavin.

To investigate at the phenotypic level the AMR pattern of the GM strain, antimicrobial susceptibility profiles were determined and compared to that of the wild-type strain (Table 5). The GM *B. subtilis* 2014-3557 had acquired phenotypic resistance to clindamycin, chloramphenicol, kanamycin, tetracycline, erythromycin, and streptomycin. This coincides with what we expected based on the genotypic results except for the susceptibility to penicillin.

**Table 5:** Minimum inhibitory concentration (MIC) of wild-type *B. subtilis* 168 and GM *B. subtilis* 2014-3557.

antibiotic class	antibiotic	*B. subtilis* 168	*B. subtilis* 2014-3557	Genes affecting resistance in *B. subtilis* 2014-3557
		mg/L	mg/L	
Lincosamides	Clindamycin	1	**>4**	*erm(B)*
Tetracycline	Tetracycline	4	**>16**	*tet(L)*
Ansamycins	Rifampicin	0.25	0.06	
Aminoglycoside	Streptomycin	8	**>32**	*rpsL* (A > G substitution)
Fusidic acid	Fusidate	1	<=0.5	
Beta-lactam	Penicillin	<=0.12	<=0.12	*blaTEM-116*
Phenicol	Chloramphenicol	<=4	**>32**	*cat(pC194)*
Aminoglycosides	Kanamycin	<=4	**8**	*aadD*
Tiamulin	Tiamulin	**>4**	**>4**	inherent
Streptogramin	Quinupristin/dalfopristin	4	2	
Glycopeptide	Vancomycin	<=1	<=1	
Aminoglycosides	Gentamicin	<=1	<=1	
DHFR inhibitor	Trimethoprim	<=2	<=2	
Macrolide	Erythromycin	<=0.25	**>8**	*erm(B)*
Quinolone	Ciprofloxacin	<=0.25	<=0.25	
Cephamycin	Cefoxitin	2	0.5	
oxazolidinone	Linezolid	<=1	<=1	
carboxylic acid	Mupirocin	<=0.5	0.5	
Sulfonamides	Sulfamethoxazole	<=64	<=64	

In bold are the values that according to the literature^63^ are considered resistant to the respective antibiotic. The > symbol is used if at the maximum concentration of an antibiotic, no bacterial growth could be detected. In the last column are the mutations and genes responsible for the phenotypic resistance.

## Discussion

In the current study, we used an unauthorized GM *B. subtilis* (2014-3557, RASFF 2014.1249) as a case study to deliver a proof of concept for a WGS strategy to fully characterize the present AMR genes and their genomic location. Our approach consisted of the combined use of short and long sequencing reads, complemented with targeted down-stream verification analyses. While this hybrid assembly approach was already successfully applied to wild-type isolates^10–13^, it had not been tested yet on GMMs that can be more complex.

We have chosen a GMM as a case study, as AMR genes are being used as selection markers during the engineering process of making the bacterium able to produce specific food and feed additives by fermentation. Often this engineering involves the use of plasmids. Before applications of GMM can be brought to the European market, the microorganism used as producer organism should be characterized and this evaluation should be presented to EFSA for authorization^14^. Recently, EFSA has highlighted the importance of including data on the full length AMR genes present in the producer strain, and their respective location (chromosome or plasmid)^14,15,33^. Additionally, the final commercialized food and feed products should be free of producer GM strains (2001/18/EC; 1829/2003/EC; 1830/18/2003)^21–23^. Enforcement laboratories are currently using qPCR methods, targeting short DNA fragments, to detect unexpected contaminations of GMMs in food and feed additives. However, due to the confidentiality of the sequence data of GMMs, it is complicated to develop specific assays. WGS is an open approach contributing to the characterization of unauthorized and/or unknown GMMs, as based on this information, specific qPCRs can be developed^27,29^. Nevertheless, qPCRs will not allow to detect full-length AMR genes, nor the determination of the location of the AMR gene, while this information is necessary for the risk assessment of the transmissibility of the AMR gene, as stated by EFSA^33^. If a viable isolate can be retrieved from the food or feed additive, our sequencing strategy would allow full characterization of the GMM, including an overview of full-length AMR genes and their location, to support the official control by the competent authorities, especially in view of the potential risk of AMR spread to the environment and/or consumers. We have chosen this particular GM *B. subtilis* 2014-3557 as a case study as there have been some previous attempts reported on the characterization of this GMM, including the presence of AMR genes, using short read sequencing^27–29^. One of these studies even indicated the presence of multiple plasmids containing several AMR genes^29^, making this an interesting yet challenging case study to deliver a proof of concept of our strategy.

We used accurate MiSeq short sequencing reads and backbone-supporting MinION long sequencing reads to perform a *de novo* hybrid assembly of the GM *B. subtilis* 2014-3557 genome. In contrast to what was previously reported using solely short sequencing reads^27–29^, where they found 4 plasmids (no published chromosome assembly)^29^ or were unable to reconstruct the chromosome and plasmid(s) (only contigs reported)^27,28^, we could assemble the full chromosome of GM *B. subtilis* 2014-3557 and an extra-chromosomal plasmid of 38 kb. This plasmid contains the entire and a partial *ribDEAHT* operon originating from *B. subtilis*, which is deleted in the chromosome. In the chromosome, 5 deletions and 2 insertions have been detected in the current characterization, of which 4 deletions and the *cat* gene insertion have already been described previously^29^. The not yet reported insertion consisted of several GM plasmids integrated in the chromosome in a very repetitive structure of at least 53 kb. These plasmids contain partial and full *ribDEAHT* operons originating from *B. amyloliquefaciens*. This result could only be obtained based on the combination of short and long read sequencing technologies. The improvement in the characterization of the GM *Bacillus* compared to previous publications^27–29^, indicates the necessity of hybrid assemblies when analysing genomes with repetitive regions. The genetic modifications facilitate the overproduction of riboflavin and AMR genes were used as markers for selection. There have been some descriptions about GM bacilli overproducing riboflavin in older patents and the scientific literature^34,35^. However, there was no sequence information available, making it difficult to do a direct comparison between *B. subtilis* 2014-3557 and earlier reports. Moreover, in the supplementary information of Paracchini *et al*.^29^ the claims of the Chinese producer company about the unauthorized GMM are described. Our current assembly of *B. subtilis* 2014-3557 confirms that genetically modified pUC19 plasmids were inserted into the chromosome. However, there was still a difference in the copy number of the inserted plasmids. The Chinese company claimed to have 5 pUC19 plasmids inserted, however in our *de novo* assembly up to 6 inserted pUC19 sequences could be detected. Moreover, it was claimed that only the inserted plasmids contained the tetracycline resistance gene. However, in our assembly, this gene was found in the chromosome (inherent to *B. subtilis* 168 genome) downstream of the 53 kb insertion and a different variant in the extra-chromosomal GM plasmid of 38 kb.

In this study, we have also evaluated assemblies made only with long MinION and PacBio reads (SPAdes^36^, Canu^37^, Miniasm^38^). However, despite the high coverage of the erroneous long reads (10–20% error rate), this resulted in fragmented assemblies and for some AMR genes this also resulted in detection of the incorrect variant. For the PacBio reads, the HGAP4 pipeline gave more accurate and contiguous results, however due to its optimization for the assembly of chromosomes, it was not possible to retrieve the complete pGMrib plasmid sequence, even with additional adjustments. Nevertheless, PacBio sequencing proved useful as extra confirmation for the hybrid assembly made with MiSeq and MinION reads. Additionally, the errors present in long read MinION assemblies can be resolved by the use of accurate short read sequencing.

Despite that the overall structure of our characterization of GM *B. subtilis* 2014-3557 is different compared to what was previously reported^29^, similar genes and mutations have been detected in both assemblies. Thus, the qPCR methods that are used by the enforcement laboratories for the detection of this GMM are still valid. However, our assembly shows that some of the primers (assays 690, 691 and 804) that were thought to bind to plasmids actually bind to the chromosome at the location where there is an insertion of these plasmids. This also explains why in the previous characterization^29^ only pGMsub03 and pGMsub04 were lost in the LHL lab by not culturing them with the appropriate antibiotics. pGMsub01 and pGMsub02 were inserted in the chromosome, thus they were not as easily lost as the 38 kb GM plasmid (which contains sequences from both pGMsub03 and pGMsub04). These qPCR methods are highly needed, as in 2018, there was a new RASFF alert concerning a GM *B. subtilis* overproducing vitamin B2 found in a feed additive (2018.2755). It is likely that *B. subtilis* 2018.2755 shares several elements with *B. subtilis* 2014-3557 such as the *rib* operon localized in a plasmid and the *cat* gene insertion based on the qPCRs that tested positive^33^. However, with all information available it is not possible to determine if it is exactly the same GM *B. subtilis strain*. Moreover, without the full characterization it is possible that cases are determined as different if a plasmid is lost during culturing or in the environment. If MiSeq reads of the 2018.2755 RASFF alert would be publicly available, it would be possible to do a de novo assembly of this isolate. Then an alignment between the short read assembly of *B. subtilis* 2018.2755 and our hybrid assembly of *B. subtilis* 2014-3557 could be made. Based on this alignment it could be determined if new sequences are present. If the latter is the case, additional long read sequencing would be necessary to accurately determine the differences. Additionally, even if the alignment would suggest that *B. subtilis* 2014-3557 and *B. subtilis* 2018.2755 are identical, then long read sequencing could be beneficial to elucidate whether there are any duplications or structural rearrangements. Alternatively, based on our assembly, it would be advisable to determine copy number differences of qPCR-558 and qPCR-804 in suspected GM *B. subtilis* overproducing riboflavin cases. If the Cq difference is much higher or lower than expected, then it would suggest that pGMsub02 is not integrated in the chromosome or integrated with more/less copies. However, with this approach no conclusions can be made about modifications in regions outside the ones targeted by these qPCR assays.

With the *de novo* hybrid assembly, it was possible to determine the correct variant and the exact location of the AMR genes on the chromosome and plasmid. All detected AMR genes conferred phenotypic resistance except for *blaTEM-116*. However, in other *Bacillus* species it has been found that some beta-lactamase genes are not expressed^39^. Based on the phenotypical data, the GM *B. subtilis* acquired resistance to erythromycin, kanamycin, and streptomycin that are critically important antibiotics and resistance to chloramphenicol, clindamycin and tetracycline that are highly important antibiotic for human medicine, according to the latest WHO publication^30^. Even more worrisome is that the genes for tetracycline and erythromycin resistance are present on an extra-chromosomal plasmid, which increases the risk of environmental spread of these AMR genes. With the improved insight into the location of the AMR genes, additional explanation on the observed AMR can be given. Indeed, while both the wild-type and the GM *B. subtilis* contain the same *tet(L)* gene in the chromosome, the GM *B. subtilis* also has a different variant of *tet(L)* localized on a plasmid that has a higher copy number (3×, as estimated based on the average coverage of MiSeq reads) than the chromosome, which likely contributed to the observed much higher MIC. The increase in MIC to clindamycin and erythromycin can be attributed to the *erm(B)* gene, while the *aadD* and *cat* genes are responsible for the resistance to kanamycin and chloramphenicol, respectively. While *aadK* confers resistance to streptomycin, this gene is also present in the wild-type *B. subtilis* 168. Therefore, the streptomycin resistance in GM *B. subtilis* 2014-3557 can likely be attributed to the chromosomal mutation in the *rpsL* gene (A > G substitution at position 129,868), which was also found by Paracchini *et al*.^29^. The *rpsL* gene encodes the ribosomal protein S12 and mutations in this gene have been associated with streptomycin resistance^32^. A bleomycin gene was detected in the GM *B. subtilis* and while bleomycin is not used in human medicine for antibacterial treatments, it has been shown that this compound, which hinders cell division, has antimicrobial effects towards wild-type *B. subtilis* that lack this resistance gene^40^. Moreover, bleomycin has been present in other GMMs^41^, therefore it is likely used as a selection marker.

In conclusion, in this study we delivered a proof of concept of a WGS strategy to successfully fully reconstruct plasmids and chromosomes, and determine the exact location of the present AMR genes. In our specific case study, our *de novo* hybrid assembly consisting of MiSeq short sequencing reads and MinION long sequencing reads improved the characterization of an unauthorized GM *B. subtilis* (RASFF 2014.1249), thereby identifying the presence and genomic location of full AMR genes, many of which are critically important, and other determinants important for the characterization of a GMM, as requested by EFSA^14,15,33^. For the most complete assembly of the chromosome and plasmid including all correct AMR genes/variants, we indeed recommend to perform hybrid assemblies. To limit the cost, it is advised to apply the WGS strategy stepwise. Thus, first MiSeq sequencing should be performed to detect all unnatural associations, AMR genes and plasmid replicons. Then a *de novo* assembly created with the MiSeq data should be compared to references^42^, e.g. the one now provided for GM *B. subtilis* 2014-3557. If this comparison shows many unnatural associations and/or AMR genes that have not been found before in related GMMs or wild-types, then it is advisable to perform long-read sequencing to fully characterize the GMM isolate. However, duplications and structural rearrangements will likely not be detected with MiSeq reads only. Therefore, for very prevalent and high impact GMM cases it would still be advisable to do additional long read sequencing even if they show high similarity to existing references of GMMs. In our case study we obtained better results with the MinION reads, i.e. longer average read lengths. An additional advantage of the portable MinION flowcells is that they are more accessible for most labs compared to PacBio sequencing.

However, our approach implies that an isolate could be obtained from the food or feed additive. If this were not the case, our strategy would need to be tested and eventually modified to be performed in a metagenomics set-up. The characterization of full AMR genes is not only important in GMM detection and identification^14^, but it is also of interest for the surveillance of pathogens, usually having a less complex genome. Therefore this study not only contributes to the EFSA demand of better characterization of GMMs, but also to the strategies to be used to strengthen our knowledge and understanding of AMR, one of the five objectives of the Global Action Plan on AMR, launched by the WHO^2^.

## Material and Methods

### Bacterial isolates

The bacterial strain used in this study is a GM *B. subtilis* strain (2014-3557) that was isolated from imported vitamin B2 80% feed additive powder and analysed by the French GMO-Laboratory “Service commun des Laboratoires” in the framework of the Rapid Alert System for Food and Feed (RASFF) 2014.1249^27,28^.

In addition, as a reference, the lab strain *B. subtilis* 168 (Ehrenber 1835, Cohn1872 AL) was purchased from the Belgian Co-ordinated Collections of Micro-organisms (BCCM) collection.

### Bacterial growth, DNA extraction and quality control of DNA

The bacterial strain was first grown on Nutrient agar at 30 °C for 48 to 72 hours with the antibiotic erythromycin. The total DNA of the *B. subtilis* strain 2014-3557 (genomic and plasmid) was extracted from the pellet of 6 ml of a 48 hour culture grown in Brain-Heart Infusion (BHI) broth at 30 °C with the antibiotic erythromycin, using the Genomic-tip 100/G according to the manufacturer’s instructions (Qiagen Benelux B.V., Venlo, the Netherlands). A Nanodrop 2000 device was used to determine the purity of the DNA. The concentration of the DNA was determined with a Qubit 3.0 fluorometer using the Broad range kit (Invitrogen, ThermoFisher Scientific). The 4200 Tapestation with genomic screentapes (Agilent) was used to determine the fragment length of the DNA. The presence of the GM plasmids was confirmed by the qPCRs from Barbau-Piednoir *et al*. and Paracchini *et al*.^27,29^ (supplementary information, Table S2).

### Whole genome sequencing

Short read sequencing libraries were prepared with an Illumina Nextera XT DNA Library Preparation Kit and sequenced on an Illumina MiSeq instrument with a 250-bp paired-end protocol (MiSeq v3 chemistry) according to the manufacturer’s instructions. Trimming of the short reads was performed with Trimmomatic (version 0.32). First the Illuminaclip option was used to remove the Nextera adapter sequences. Then a sliding window approach of four bases and trimming when the Phred score dropped below 30 was employed. Lastly, the leading and trailing bases of a read were removed when the Phred score dropped below 3^43^. After trimming, this amounted to 920,796 reads with an average Phred-score > 30. These statistics from the reads were extracted with FastQC (version 0.11.7). Based on mapping results, the MiSeq reads amounted to a coverage of 49 × (SD 17) (Fig. S1A).

A long read MinION sequencing library was prepared by using the 1D ligation sequencing kit (SQK-LSK108, Oxford Nanopore) according to the manufacturer’s protocol for genomic DNA (version 6). The optional steps of shearing the DNA to 8 kb fragments with Covaris G tubes and addition of the control DNA (a 3.6 kb fragment of Lambda phage) were included, while the DNA repair was not performed. The sequencing was carried out on a R9.4 flowcell (Oxford Nanopore) and sequenced for 48 hours, which produced 0.25 million reads, a N50 of 9452, average read size of 7,731 bp and a coverage of 343 ×(SD 74) (Fig. S1C). Local basecalling was performed with Guppy (version 3.1.5) (Oxford Nanopore Technologies) with the option enabled to trim the sequencing adapters. Then NanoFilt (version 2.0.0)^44^ was used to ensure that only the reads with a Phred-score > 7 and a length > 1,000 were used in the *de novo* assembly. These statistics of the reads and quality scores were extracted with NanoStat (version 0.8.0)^44^. The control DNA was removed by using the NanoLyse (version 0.5.0) software^44^.

PacBio sequencing was performed on the wild-type *B. subtilis* 168 and the genetically modified *B. subtilis* 2014-3557 with the PacBio Sequel at Baseclear (Leiden, the Netherlands). This run produced 1,649,115 reads with an average size of 4,347 bp and coverage of 1,657 ×(SD 376) (Fig. S1E). These reads were used in the HGAP4 pipeline (SMRTlink version 6.0.0.47841) from PacBio Sequel^45,46^.

### Bioinformatics analysis

*De novo* hybrid assembly with MiSeq and MinION reads was carried out using Unicycler (version 0.4.8) at default parameters^47^. The following tools were used in the Unicycler pipeline: SPAdes (version 3.7.1), Miniasm (version 0.3), Racon (version 1.4.3), makeblastdb (version 2.7.1+), tblastn (version 2.7.1+), bowtie2 (version 2.3.4.3), Samtools (version 1.9), java (version 1.8) and Pilon (version 1.22)^36,38,48–52^. *De novo* assemblies with other software (SPAdes hybrid, Canu and Miniasm) were performed and corrected with Racon^48^ and Pilon^52^. Then circularity of the contigs was determined with Berokka (version 0.2.1)^53^. However, the *de novo* assembly obtained from Unicycler was determined to be the most accurate and was used for all subsequent analyses (Table S3). Visualization of the Unicycler assembly was performed in Bandage (version 0.8.1)^54^ (data not shown). Visualization of the integrated region and plasmids was performed with SeqBuilder (version 10.0.1)^55^. ResFinder^3^ was used to determine genes responsible for the genotypic AMR resistance. PlasmidFinder^56^ was used for the detection of plasmid replicons. BWA-MEM (version 0.7.12-r1039) was used for the mapping of MiSeq reads. Minimap2^57^ was used for mapping the MinION and PacBio reads to the reference genome and hybrid assembly. SAMtools (version 1.9) was used for processing SAM files and extracting mapping statistics^51^. MauveProgressive (version 2.4.0) was used to compare the reference genome to the *de novo* assembly^58^. IGV (version 2.4.4) and Qualimap (version 2.2.1) were used to visualize the mapping, coverage, GC content and the mapping quality^59,60^. Annotations were performed with Prokka (version 1.13.3)^61^.

### (q)PCR

In each (q)PCR reaction, DNA extracted from the wild-type *B. subtilis* 168 was used as a negative control. All PCR reactions developed in this study were created by use of the primer-blast software^62^. This software was also used to test *in silico* if it was possible to produce any aspecific products in either the GMM or wild-type. For the sequence of all primers, see the supplementary information (Table S1).

The conventional PCRs with amplicons of <1000 bp were performed in a final reaction volume of 25 μl containing 1x DreamTaq Master Mix (Thermo Fisher Scientific), 300 nM of forward and reverse primer and 5 μl DNA template (1 ng/μl). The PCR cycle program consisted of a denaturation step at 95 °C for 3 minutes, followed by 35 cycles of 30 seconds at 95 °C, 30 seconds at 55 °C and an extension step at 72 °C for 1 minute. At the end, 1 cycle at 72 °C of 15 minutes was performed.

The long range PCRs with amplicon sizes of 9–10 kbp were performed in a final reaction volume of 25 μl containing 1x Master Mix (KAPA readymix), 300 nM of forward and reverse primer and 5 μl DNA template. The PCR cycle program consisted of a denaturation step at 95 °C for 3 minutes, followed by 35 cycles of 30 seconds at 95 °C, 30 seconds at 55 °C and an extension step at 72 °C for 1 minute (+1 minute per kb of amplicon size). At the end, 1 cycle at 72 °C for 1 minutes (+1 minute per kb of amplicon size) was performed.

qPCRs from Barbau-Piednoir *et al*. and Paracchini *et al*. were used under the same conditions as described in their respective publications and performed in triplicate using 5 ng template input DNA^27,29^. A copy number variation analysis between two qPCR reactions was done with the following formula: copy number difference = 2^ΔCq^.

### Antimicrobial susceptibility

The antimicrobial susceptibility profiles (clindamycin, tetracycline, rifampicin, streptomycin, fusidate, penicillin, chloramphenicol, kanamycin, tiamulin, quinupristin/dalfopristin, vancomycin, gentamycin, trimethoprim, erythromycin, ciprofloxacin, cefoxitin, linezolid, mupirocin and sulfamethoxazole) of the GM-*Bacillus* strain 2014-3557 and the model organism *B. subtilis* 168 were determined using the microdilution method involving the Sensititre Staphylococci plate – EUST (Veterinary Reference Card, Thermo Scientific). For each of the tested antimicrobials, the minimal inhibitory concentration (MIC) was determined. The MIC is the lowest concentration of an antimicrobial (in mg/l) that inhibits the visual growth of a micro-organism under the defined *in vitro* conditions and after a specified time period. There are no MIC values for *Bacillus* for this antimicrobial available with EUCAST (the European Committee on Antimicrobial Susceptibility Testing). Therefore, the MIC values from EFSA were used to determine whether there was phenotypic resistance against the antibiotics that were used^63^.

### Dosage of riboflavin (vitamin B2)

The riboflavin concentration was determined in the supernatant of the GM-*B. subtilis* strain (2014-3557) and that obtained from wild-type *B. subtilis* (food origin) using an HPLC method^64^; Standard (−)-riboflavin (Sigma-Aldrich R7649); Column Lichrospher 60 RP Select B (5 µm) 250 × 4 mm (Merck 1.50984.0001) in column oven Igloo-Cil with column temperature at 30 °C; Isocratic pump Varian prostar 220 at flow rate 0,7 mL/min with mobile phase methanol/sodium acetate 0,05 M buffer 40/60 v/v; Automatic injector Varian prostar 410 with injection volume at 30 µL; Fluorimetric detector Varian prostar 363 at superlow wide mode with λ excitation at 422 nm and λ emission at 522 nm). As blank, BHI culture medium was included. The dosage was performed on the supernatant of 50 ml cultures (centrifuged at 5000 g during 10 min) after 48 hours of growth in BHI medium at 30 °C (Mac Farland 0.8). For each strain, 4 repetitions were included and each supernatant was analysed in duplicate. The blank was analysed only once.

## Supplementary information

Supplementary Information

## Data Availability

Raw sequencing data and the *de novo* hybrid assembly were submitted to NCBI Sequence Read Archive (SRA)^65^ and NCBI Genbank^66^ under the accession number PRJNA576869.
