# Supplementary material for: Combining short and long read sequencing to characterize antimicrobial resistance genes on plasmids applied to an unauthorized genetically modified Bacillus
Source: Sci Rep. 2020 Mar 9;10:4310. doi: 10.1038/s41598-020-61158-0 (PMC7062872; doi:10.1038/s41598-020-61158-0)
Supplement: Supplementary file 1 — Supplementary Information [file 41598_2020_61158_MOESM1_ESM.pdf]

Supplementary information

Title

Combining short and long read sequencing to characterise antimicrobial resistance genes on plasmids applied to an unauthorized genetically modified *Bacillus*

Authors

Bas Berbers[1, 2], Assia Saltykova[1, 2], Cristina Garcia-Graells[3], Patrick Philipp[4], Fabrice Arella[4], Kathleen Marchal[2, 5], Raf Winand[1], Kevin Vanneste[1], Nancy H. C. Roosens[1£], Sigrid C.J. De Keersmaecker[1*£]

[1]Transversal activities in Applied Genomics, Sciensano, Brussels, Belgium; [2]Department of Information Technology, IDLab, Ghent University, IMEC, Ghent, Belgium; [3]Foodborne Pathogens, Sciensano, Brussels, Belgium, [4]Service Commun des Laboratoires, Illkirch-Graffenstaden, France, [5]Department of Plant Biotechnology and Bioinformatics, Ghent University, Ghent, Belgium.

* = corresponding author (Sigrid.DeKeersmaecker@sciensano.be); £ = equal contribution

Table S1: (q)PCRs tested the wild type *B. subtilis* 168 and GM *B. subtilis* 2014-3557, their targets and primer/probe sequences

target	name	Oligonucleotide sequence (5'-3')	Reference
**qPCR**			
junction of rib operon-fragment to pSM19035	VitB2-UGM-F	GATACCAAACGAAATGGGACAT	Barbau-Piednoir et al. 2015
	VitB2-UGM-R	TTCAGCGTAACAGACATAATTTTT	
	VitB2-UGM-P	1ICCGIGIAGGAAI111IAGAGIAGAA	
Integration site of *cat* in *B. subtilis*	558-F	CGAGCTTTTGCGCGTATA	Parachini et al. 2017
	558-R	GCCATTCCAATACAAAACCACATA	
	558-probe	CGGATCTAACGCATGCTCCGCA	
Plasmid pGMBsub01 (junction of pUB110 to pUC19)	690-F	GATGAATTATATCAACATATTAAGCCTTTGG	Parachini et al. 2017
	690-R	GCTATGACCATGATTACGCCAAG	
	690-probe	AAGATCCGGGGAATTGCTGCAGG	
Plasmid pGMBsub01 (junction of B. amyloliquefaciens rib-operon to pUC19)	691-F	CGATTAAGTTGGGTAACGCCA	Parachini et al. 2017
	691-R	TTCTCTAAAGAAAACTGCTCGTACG	
	691-probe	ACGGCCAGTGAATTCGCAAGACG	
Plasmid pGMBsub02 (junction of deleted B. amyloliquefaciens rib-operon)	804-F1	AGACCGCGTTTACAGTCAGCAT	Parachini et al. 2017
	804-R1	CTCGAATTCTTTTTTCGTTCCAA	
	804-probe	ACCACAAGCTGACCGAATATGCGGAT	
Plasmid pGMBsub03 (junction of 3'-ribfragment to pUC19)	693-F	TCGTGCACAGCTTGAAATCTAGA	Parachini et al. 2017
	693-R	GGAAACAGCTATGACCATGATTACG	
	693-probe	CCTCTAGAGTCGACCTGCAGGCATGC	
Plasmid pGMBsub04 (junction of rib operon-fragment to pSM19035)	694-F	CATTCGATTGTGCGAGCG	Parachini et al. 2017
	694-R	TGGTATTTTTTGTATTCAGCGTAACAGACATAAT	
	694-probe	CAGGCGAATTCCAGTTAAATTCCGTGTAGG	
**PCR**			
Integration of GM plasmids in chromosome 5' side	PCR-intp1-F	AAGATGCCCCTACGACAACG	This study
	PCR-intp1-R	GCTCCTGTTCAATGAGGACCA	
Integration of GM plasmids in chromosome 3' side	PCR-intp2-F	AATGGTTGCCACGGGTATGT	This study
	PCR-intp2-R	GATGACGTCAGTCCGATCCC	
unique region within integration of GM plasmids in chromosome	PCR-longrange-1-F	GCATATTCGGTCAGCTTGTGGT	This study
	PCR-longrange-1-R	CGTTCGCGTTCCATGATGAC	
	PCR-longrange-2-F	GCATATTCGGTCAGCTTGTGGT	This study

unique region within integration of GM plasmids in chromosome	PCR-longrange-2-R	GAGGCGCAACGACACATTAC	
No chromosomal integration of GM plasmids	PCR-longrange-3-F	GATGACGTCAGTCCGATCCC	This study
	PCR-longrange-3-R	GCCTGTAAGGACTCAAGCGT	

Table S2: Cq values of *B. subtilis* 168 and *B. subtilis* 3557 from qPCRs developed by Barbau-Piednoir et al. 2015 and Paracchini et al. 2017.

qPCR assay	total input DNA	Cq *B. subtilis* 168	Average Cq *B. subtilis* 3557	Cq difference compared to 558	calculation copy number difference*	expected copy number difference
558	5 ng	N/A	18.38 (SD 0.02)			
804	5 ng	N/A	16.49 (SD 0.06)	1.89 (SD 0.06)	3.71 (SD 0.15)	4
690	5 ng	N/A	15.03 (SD 0.06)	3.35 (SD 0.06)	10.20 (SD 0.41)	6
691	5 ng	N/A	14.89 (SD 0.13)	3.49 (SD 0.13)	11.24 (SD 1.03)	6
693	5 ng	N/A	16.25 (SD 0.01)	2.13 (SD 0.02)	4.38 (SD 0.05)	3
694	5 ng	N/A	16.48 (SD 0.05)	1.90 (SD 0.05)	3.73 (SD 0.14)	3
vitB2-UGM	5 ng	N/A	16.44 (SD 0.08)	1.94 (SD 0.08)	3.84 (SD 0.21)	3

*These qPCR reactions were initially only performed to confirm that the plasmids that they should detect, were still present in the DNA extracts and not lost during the culturing. Theoretically, the copy number of the targeted regions could be calculated based on the obtained Cq values, under the assumption that the qPCR efficiency is each time 100%. However, the qPCR efficiency of the assays is unknown. Additionally, the precision of a qPCR assay can range between +/- 1 Cq of the obtained value. Therefore, it is expected that the calculated copy number difference, as indicated in the table above, does not exactly match with the expected copy number difference. Alternatively, the obtained values may indicate that the integrated region (with repetitive plasmids) is larger than 53 kb. However, the currently available methods (including sequencing technologies) do not allow to determine this. Nevertheless, this will not impact the results of this study, which aimed to characterize the present AMR genes and their location (chromosome or extra-chromosomal plasmid).

Table S3: De novo assembly statistics of genetically modified *Bacillus subtilis* 2014-3557.

**Parameter**	**Value**
**Assembly from Unicycler ***	
Contigs	2
Total size (bp)	4,279,307
N50	4,240,660
AMR genes detected with ResFinder (100% identity)$	11/11
Contig 1 size (bp)	4,240,660
Circularity contig 1	Yes
Contig 2 size (bp)	38,647
Circularity contig 2	Yes
GC (%)	43.53%
**Assembly from Spades****	
Contigs	44
Total size (bp)	4,240,331
N50	2,234,155
AMR genes detected with ResFinder (100% identity)$	9/11
Longest contig (bp)	2,234,155
GC%	43.53%
**Assembly from Canu**,%**	
Contigs	2
Total size (bp)	4,265,747
N50	4,227,093
AMR genes detected with ResFinder (100% identity)$	9/11 (ErmB = 99.73% sequence identity)
Contig 1 size (bp)	4,227,093
Circularity contig 1	Yes
Contig 2 size (bp)	38,654
Circularity contig 2	Yes
GC%	43.52%

**Assembly from Miniasm**,%**	
Contigs	6
Total size (bp)	4,367,405
N50	4,225,730
AMR genes detected with ResFinder (100% identity)[$]	7/11 (aadK = 99.88% sequence identity)
Contig 1 size (bp)	4,225,730
Circularity contig 1	No
Contig 2 size (bp)	38,619
Circularity contig 2	Yes
Contig 3 size (bp)	45,880
Circularity contig 3	No
Contig 4 size (bp)	19,790
Circularity contig 4	No
Contig 5 size (bp)	7,556
Circularity contig 5	Yes
Contig 6 size (bp)	29,830
Circularity 6	No
GC%	43.56%
**Assembly from HGAP4****	
Contigs	3
Total size (bp)	4,220,468
N50	4,198,882
AMR genes detected with ResFinder (100% identity)[$]	7/11
Contig 1 size (bp)	4,198,882
Circularity contig 1	Yes
Contig 2 size (bp)	10,208
Circularity contig 2	No
Contig 3 size (bp)	11378
Circularity contig 3	Yes
GC%	43.57

* The hybrid assembly from Unicycler still gave the most accurate representation of the GMM, as evaluated based on contig size, N50, circularity of contigs, detected genes (ResFinder).

[$] Based on the expectation that some AMR genes should be found multiple times (Paracchini et al. 2017).

** Berokka was used to check for circularity of the contigs.

[%]The assemblies from Canu and Miniasm were improved by correcting them with long and short sequencing reads (Racon and Pilon).

Table S4: read and mapping statistics of the MiSeq, MinION and PacBio reads to the *de novo* assembly of the genetically modified *Bacillus subtilis* 2014-3557.

**Sequencing read statistics**	
**Parameter**	**Value**
SRA accession for MiSeq reads	SRR10260289
MiSeq average read size (bp)	233
Longest MiSeq read (bp)	250
SRA experiment accession for MinION reads	SRR10260288
MinION average read size (bp)	7,731
Longest MinION read (bp)	65,992
SRA experiment accession for PacBio	SRR10260287
PacBio average read size (bp)	4,347
Longest PacBio read (bp)	81,281
**Mapping statistics**	
**Parameter**	**Value**
MiSeq reads mapped to assembly (frequency, %)	920,419 (99.9%)
Mean coverage with MiSeq reads of complete assembly	49x (SD 17)
Mean coverage with MiSeq reads of contig 1	48x (SD 14)
Mean coverage with MiSeq reads of contig 2	139x (SD 41)

MinION reads mapped to assembly (frequency, %)	165,398 (100%)
Mean coverage with MinION reads of complete assembly	343x (SD 74)
Mean coverage with MinION reads of contig 1	339x (SD 61)
Mean coverage with MinION reads of contig 2	771x (SD 123)
PacBio reads mapped to assembly (frequency, %)	1,786,125 (98.97%)
Mean coverage with PacBio reads of complete assembly	1,657x (SD 376)
Mean coverage with PacBio reads of contig 1	1,629x (SD 215)
Mean coverage with PacBio reads of contig 2	4,770x (SD 920)

Table S5: several long reads that span the repetitive regions of the integration of pGMsub01 and pGMsub02 in the chromosome of the GM *Bacillus subtilis*.

	Read name	Read length (bp)	Alignment length (bp)	Position (start-end)	MAPQ	Sequence identity (%)
MinION	1585efa7-3977-4a86-9bd8-842155f4aef2	26,174	26142	2,391,242-2,417,383	60	89%
	de672ed4-ef9f-4cad-8173-45e963e33f94	28,342	29099	2,412,350-2,441,448	60	91%
	276f3580-7b10-438b-803e-3c4097ebab75	12,508	12864	2,438,496-2,451,359	60	93%
	d7a34c26-f268-42e2-bf38-f60a90404839	19,677	20451	2,445,567-2,466,017	60	88%
PacBio	m54072_190304_072122/26739054/68354_82072	13,718	13,421	2,398,423-2,411,843	60	93%
	m54072_190304_072122/66650401/7920_21525	13,605	12,930	2,416,885-2,429,814	60	91%
	m54072_190304_072122/58000262/34490_47824	13,334	11,720	2,434,729-2,446,448	60	85%
	m54072_190304_072122/58000262/34490_47824	14,261	14,047	2,454,697-2,468,743	60	90%

The repetitive region spans from 2406920-2459995.

Table S6: SNPs determined with MauveProgressive between *B. subtilis 168* (NZ_CP010052.1) and GM *B. subtilis* 2014-3557

SNP pattern	position *B. subtilis* 168	position in GM *B. subtilis* 2014-3557
G > A	10275	9866
T > C	12348	11939
A > G	34740	34331
G > A	90766	90357
T > C	90806	90397
G > A	91000	90591
T > C	100290	99881
C > T	116949	116540
A > G	129868	129459
A > G	147699	147290
A > G	155883	155474
T > C	163250	162841
G > T	163447	163038
T > C	163448	163039
G > A	163451	163042
C > A	166706	166297
T > C	166772	166363
T > C	168859	168450
G > C	169386	168977
T > C	173860	173452
C > T	334196	333788

G > A	374652	374243
G > T	401034	400624
C > T	495783	495374
C > T	585795	564875
C > T	625219	604299
A > G	636098	615178
G > T	701624	680701
C > T	745647	724724
G > A	774096	753173
G > A	902245	881322
G > T	914315	893392
A > G	961826	940900
C > T	981513	960587
C > T	1181848	1160933
T > G	1224533	1203611
T > A	1264293	1243371
T > A	1357583	1336661
C > T	1416165	1395243
T > G	1424651	1403729
C > T	1431623	1410701
A > G	1442381	1421459
A > G	1485965	1465043
T > G	1528068	1507146
C > T	1589023	1568101
G > A	1606431	1585509
C > T	1612142	1591220
G > A	1614767	1593845
T > A	1647417	1626495
C > A	1648161	1627239
C > G	1663517	1642594
C > T	1675861	1654938
G > A	1694872	1673949
G > A	1694910	1673987
G > C	1736834	1715911
G > A	1738441	1717518
G > A	2011103	1991463
A > G	2041111	2021471
C > T	2064509	2044869
C > T	2073554	2053914
G > A	2110812	2091172
C > T	2117669	2098029
C > T	2139578	2119938
T > C	2157751	2138111
G > A	2190621	2170981
A > G	2201421	2181781
C > T	2324956	2305316
G > A	2352542	2332902
G > A	2362018	2342378
C > T	2374230	2354590

C > T	2374233	2354593
A > T	2374242	2354602
A > G	2374295	2354655
G > T	2374296	2354656
A > G	2374309	2354669
G > A	2374311	2354671
T > A	2374336	2354696
T > G	2374338	2354698
C > T	2374340	2354700
T > G	2374344	2354704
C > A	2374353	2354713
A > G	2374368	2354728
A > G	2374371	2354731
C > T	2374376	2354736
G > A	2374386	2354746
G > A	2374389	2354749
T > C	2374392	2354752
C > T	2374398	2354758
A > T	2374424	2354784
C > T	2374456	2354816
C > T	2374509	2354869
T > C	2374557	2354917
A > G	2374587	2354950
A > T	2374596	2354959
A > G	2374608	2354971
T > G	2374673	2355036
C > T	2374683	2355046
C > T	2374691	2355054
C > T	2374692	2355055
T > C	2374711	2355074
C > T	2374727	2355090
T > C	2374755	2355118
G > A	2374770	2355133
G > A	2374795	2355158
C > T	2374812	2355175
C > T	2374827	2355190
A > T	2374843	2355206
G > A	2374850	2355213
A > G	2374851	2355214
C > T	2374863	2355226
G > A	2374884	2355247
T > G	2374890	2355253
C > T	2374972	2355335
C > T	2374975	2355338
A > G	2375005	2355368
T > C	2375008	2355371
A > T	2375020	2355383
A > C	2375029	2355392
C > T	2375032	2355395

C > T	2375044	2355407
T > C	2375099	2355462
A > G	2375101	2355464
G > A	2375104	2355467
C > T	2375113	2355476
A > T	2375116	2355479
G > C	2375121	2355484
T > G	2375162	2355525
G > C	2375167	2355530
C > T	2375182	2355545
T > C	2375194	2355557
A > G	2375266	2355629
A > G	2375272	2355635
G > A	2375313	2355676
A > G	2375327	2355690
A > G	2375329	2355692
A > G	2375350	2355713
T > C	2375362	2355725
A > G	2375365	2355728
G > A	2375374	2355737
C > T	2375380	2355743
A > G	2375383	2355746
G > A	2375388	2355751
C > T	2375398	2355761
T > C	2375410	2355773
C > T	2375413	2355776
A > T	2375422	2355785
T > C	2375428	2355791
G > A	2375452	2355815
A > G	2375455	2355818
C > T	2375461	2355824
T > C	2375487	2355850
A > T	2375527	2355890
A > G	2375529	2355892
G > A	2375538	2355901
T > A	2375540	2355903
A > G	2375590	2355953
C > T	2375605	2355968
A > C	2375608	2355971
A > T	2375611	2355974
C > T	2375617	2355980
C > T	2375632	2355995
G > T	2375635	2355998
G > A	2375656	2356019
C > T	2375671	2356034
T > G	2375674	2356037
A > G	2375692	2356055
T > C	2375694	2356057
T > C	2375723	2356086

T > C	2375734	2356097
C > T	2375740	2356103
A > G	2375746	2356109
C > T	2375751	2356114
A > T	2375752	2356115
C > T	2375770	2356133
T > A	2375788	2356151
C > A	2375797	2356160
C > G	2375800	2356163
A > T	2375815	2356178
T > C	2375817	2356180
C > T	2375829	2356192
C > T	2375830	2356193
T > C	2375850	2356213
A > T	2375854	2356217
C > T	2375861	2356224
G > T	2375872	2356235
C > T	2375903	2356266
A > G	2375933	2356296
A > C	2376020	2356383
G > T	2376029	2356392
A > G	2376068	2356431
C > G	2376074	2356437
C > A	2376104	2356467
C > T	2376113	2356476
A > G	2376116	2356479
A > G	2376125	2356488
C > G	2376131	2356494
T > G	2376134	2356497
T > C	2376164	2356527
G > A	2376182	2356545
G > T	2376194	2356557
T > A	2376197	2356560
C > T	2376212	2356575
A > G	2376227	2356590
A > G	2376248	2356611
T > C	2376259	2356622
G > A	2376284	2356647
T > C	2376302	2356665
A > G	2376311	2356674
A > T	2376344	2356707
G > A	2376365	2356728
T > C	2376374	2356737
A > G	2376383	2356746
A > G	2376398	2356761
G > C	2376428	2356791
C > T	2376437	2356800
C > G	2376440	2356803
C > T	2376444	2356807

C > T	2376446	2356809
A > C	2376455	2356818
G > T	2376456	2356819
G > A	2376461	2356824
A > G	2376467	2356830
G > T	2376470	2356833
T > C	2376503	2356866
T > C	2376515	2356878
G > T	2376517	2356880
T > G	2376524	2356887
C > T	2376530	2356893
A > T	2376564	2356927
C > G	2376566	2356929
C > A	2376590	2356953
T > C	2376614	2356977
A > G	2376629	2356992
C > A	2376637	2357000
C > A	2376647	2357010
C > T	2376649	2357012
T > C	2376665	2357028
A > G	2376667	2357030
A > G	2376671	2357034
C > T	2376674	2357037
T > C	2376689	2357052
G > T	2376701	2357064
C > T	2376702	2357065
T > C	2376703	2357066
G > T	2376707	2357070
A > G	2376731	2357094
C > T	2376734	2357097
A > G	2376736	2357099
G > A	2376779	2357142
C > T	2376803	2357166
G > A	2376821	2357184
A > T	2376836	2357199
T > C	2376844	2357207
C > T	2376854	2357217
A > T	2376855	2357218
G > A	2376869	2357232
C > G	2376878	2357241
T > C	2376892	2357255
C > G	2376901	2357264
G > T	2376906	2357269
G > A	2376941	2357304
C > T	2376956	2357319
G > T	2376998	2357361
T > C	2376999	2357362
G > T	2377010	2357373
T > A	2377012	2357375

A > G	2377018	2357381
A > G	2377052	2357415
A > T	2377094	2357457
A > T	2377100	2357463
T > C	2377103	2357466
T > C	2377118	2357481
G > A	2377133	2357496
C > A	2377139	2357502
T > A	2377148	2357511
A > T	2377167	2357530
T > C	2377173	2357536
T > C	2377177	2357540
C > A	2377181	2357544
A > T	2377187	2357550
C > T	2377189	2357552
G > A	2377190	2357553
C > T	2377193	2357556
A > T	2377195	2357558
C > T	2377204	2357567
C > T	2377210	2357573
T > C	2377220	2357583
A > G	2377226	2357589
G > T	2377256	2357619
G > T	2377263	2357626
G > A	2377274	2357637
A > G	2377310	2357673
G > T	2377339	2357702
T > C	2377340	2357703
C > G	2377373	2357736
G > A	2377381	2357744
C > G	2377396	2357759
C > A	2377405	2357768
G > A	2377406	2357769
T > G	2377415	2357778
A > T	2377422	2357785
T > A	2377426	2357789
T > C	2377483	2357846
C > T	2377485	2357848
A > G	2377513	2357876
A > C	2377555	2357919
T > C	2377600	2357963
T > C	2377637	2358000
C > T	2377681	2358044
C > T	2377684	2358047
G > A	2377690	2358053
A > G	2377693	2358056
A > G	2377694	2358057
T > C	2377714	2358077
T > C	2377721	2358084

A > T	2377723	2358086
G > A	2377732	2358095
C > G	2377747	2358110
T > G	2377762	2358125
T > G	2377783	2358146
T > G	2377807	2358170
T > C	2377816	2358179
G > A	2377840	2358203
A > G	2377843	2358206
A > G	2377864	2358227
C > A	2377866	2358229
A > C	2377882	2358245
C > A	2377888	2358251
A > T	2377921	2358284
G > T	2377923	2358286
A > G	2377924	2358287
A > T	2377957	2358320
C > T	2377987	2358350
T > C	2378087	2358450
A > G	2378088	2358451
C > T	2378103	2358466
A > G	2378121	2358484
A > G	2378142	2358505
T > C	2378171	2358534
G > T	2378175	2358538
T > A	2378196	2358559
C > T	2378200	2358563
G > A	2378202	2358565
A > G	2378217	2358580
T > A	2378227	2358590
G > A	2378235	2358598
T > C	2378250	2358613
T > C	2378268	2358631
A > G	2378269	2358632
A > T	2378271	2358634
T > C	2378304	2358667
C > T	2378337	2358700
A > G	2378340	2358703
C > A	2378352	2358715
C > T	2378379	2358742
A > G	2378382	2358745
T > G	2378400	2358763
C > T	2378436	2358799
A > T	2378442	2358805
G > C	2378451	2358814
T > C	2378482	2358845
A > G	2378484	2358847
T > C	2378503	2358866
C > T	2378520	2358883

C > T	2378577	2358940
T > G	2378579	2358942
G > A	2378597	2358960
A > G	2378610	2358973
A > G	2378619	2358982
T > C	2378631	2358994
C > T	2378646	2359009
A > G	2378667	2359030
T > C	2378688	2359051
G > A	2378703	2359066
G > A	2378718	2359081
G > A	2378739	2359102
C > T	2378787	2359150
T > C	2378808	2359171
A > G	2378861	2359224
T > C	2378886	2359249
A > G	2378898	2359261
T > C	2378904	2359267
A > G	2378922	2359285
G > A	2378946	2359309
C > A	2378973	2359336
T > C	2378976	2359339
A > G	2378993	2359356
T > G	2379002	2359365
A > G	2379020	2359383
G > T	2379025	2359388
G > A	2379040	2359403
T > C	2379044	2359407
A > G	2379054	2359417
T > C	2379078	2359441
A > G	2379090	2359453
C > T	2379108	2359471
A > G	2379167	2359530
T > G	2379179	2359542
T > C	2379209	2359572
A > T	2379215	2359578
A > C	2379251	2359614
A > T	2379254	2359617
G > T	2379311	2359674
A > G	2379314	2359677
C > T	2379377	2359740
G > T	2379404	2359767
A > G	2379407	2359770
A > T	2379416	2359779
A > G	2379422	2359785
G > A	2379428	2359791
T > C	2379434	2359797
G > T	2379481	2359844
C > T	2379482	2359845

T > G	2379485	2359848
C > T	2379491	2359854
T > G	2379497	2359860
G > A	2379545	2359908
A > G	2379560	2359923
C > T	2379566	2359929
A > G	2379584	2359947
C > A	2379593	2359956
C > T	2379602	2359965
T > A	2379629	2359992
T > G	2379653	2360016
T > C	2379659	2360022
T > C	2379692	2360055
C > T	2379695	2360058
A > C	2379716	2360079
T > C	2379725	2360088
A > G	2379742	2360105
T > A	2379746	2360109
C > T	2379761	2360124
A > T	2379782	2360145
C > T	2379797	2360160
G > A	2379803	2360166
C > T	2379806	2360169
A > G	2379821	2360184
T > G	2379845	2360208
C > T	2379851	2360214
A > G	2379890	2360253
A > G	2379917	2360280
T > C	2379932	2360295
A > T	2379935	2360298
C > T	2379974	2360337
T > C	2380001	2360364
G > C	2380016	2360379
G > A	2380019	2360382
T > G	2380061	2360424
C > T	2380076	2360439
A > T	2380088	2360451
G > A	2380097	2360460
T > G	2380160	2360523
T > C	2380223	2360586
C > T	2380229	2360592
G > A	2380325	2360688
G > A	2380393	2360756
A > G	2380394	2360757
T > C	2380396	2360759
G > C	2380411	2360774
A > T	2380444	2360807
A > G	2380447	2360810
C > T	2380459	2360822

C > A	2380486	2360849
C > G	2380498	2360861
G > A	2380537	2360900
C > A	2380567	2360930
C > T	2380573	2360936
A > G	2380575	2360938
T > C	2380614	2360977
C > T	2380618	2360981
T > C	2421619	2401979
C > T	2426564	2406924
T > A	2426595	2406955
C > T	2426600	2406960
C > T	2439695	2464675
C > T	2440650	2465630
C > T	2457864	2482844
T > C	2544910	2569890
G > A	2595245	2620225
C > T	2605913	2630893
T > C	2612409	2637389
G > A	2622101	2647081
G > A	2691359	2716339
G > A	2893918	2918898
T > C	2918929	2943909
A > T	2920866	2945846
T > C	2933107	2958087
C > T	2966075	2991055
T > A	2982429	3007409
T > C	2982449	3007429
A > G	3010794	3035773
A > C	3178091	3203063
G > A	3178177	3203149
C > T	3203755	3228727
C > T	3343230	3368006
C > A	3346334	3371110
C > T	3391697	3416473
G > A	3406741	3431517
C > T	3408414	3433190
C > T	3425329	3450105
T > C	3469314	3494090
G > A	3471719	3496495
G > A	3498290	3523066
C > T	3498438	3523214
G > A	3503247	3528023
C > T	3599761	3624537
C > T	3600113	3624889
C > T	3614126	3638902
T > A	3662063	3686839
C > T	3680129	3704905
T > C	3696881	3721657

G > A	3707626	3732402
G > A	3797112	3821887
C > A	3902319	3926948
C > T	3961957	3986586
C > A	4005705	4030334
G > T	4037496	4062125
G > A	4156014	4180646
G > A	4180195	4204827
G > A	4180337	4204969

A.

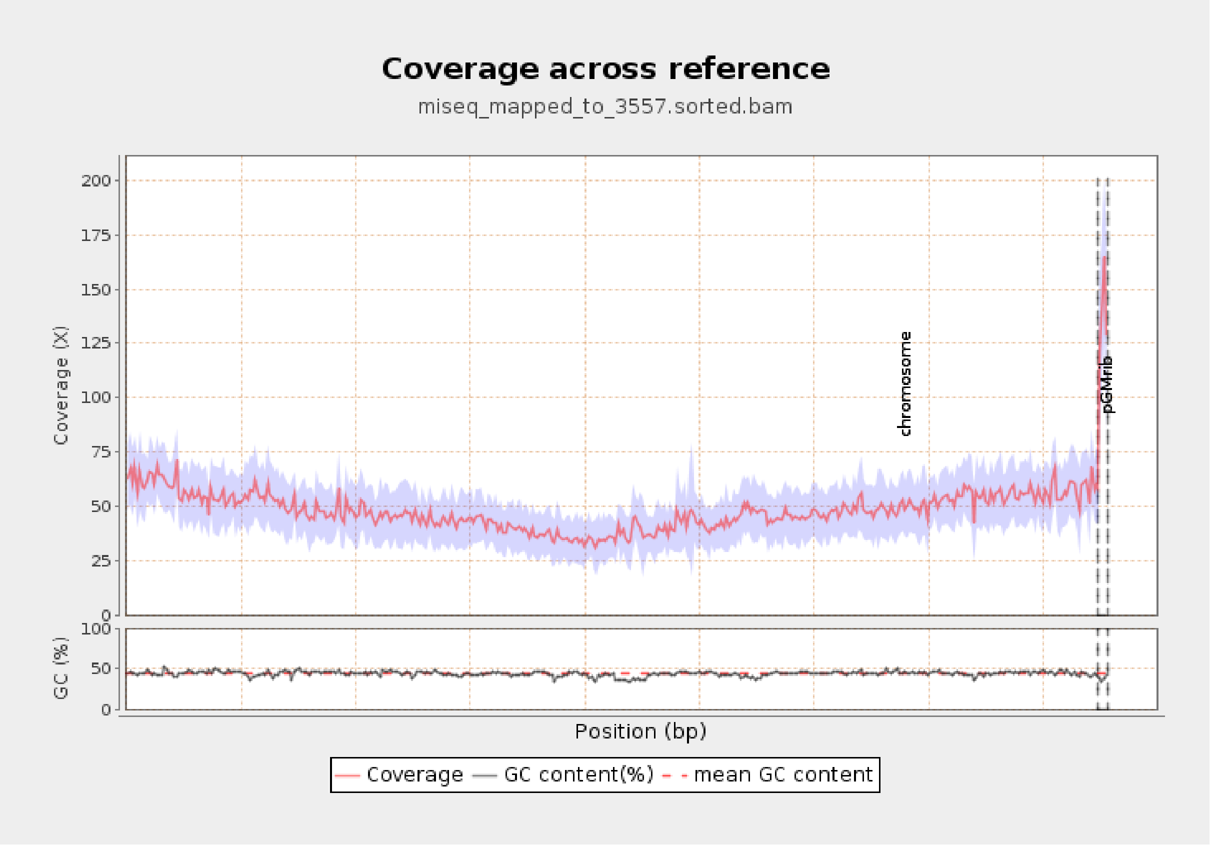

B.

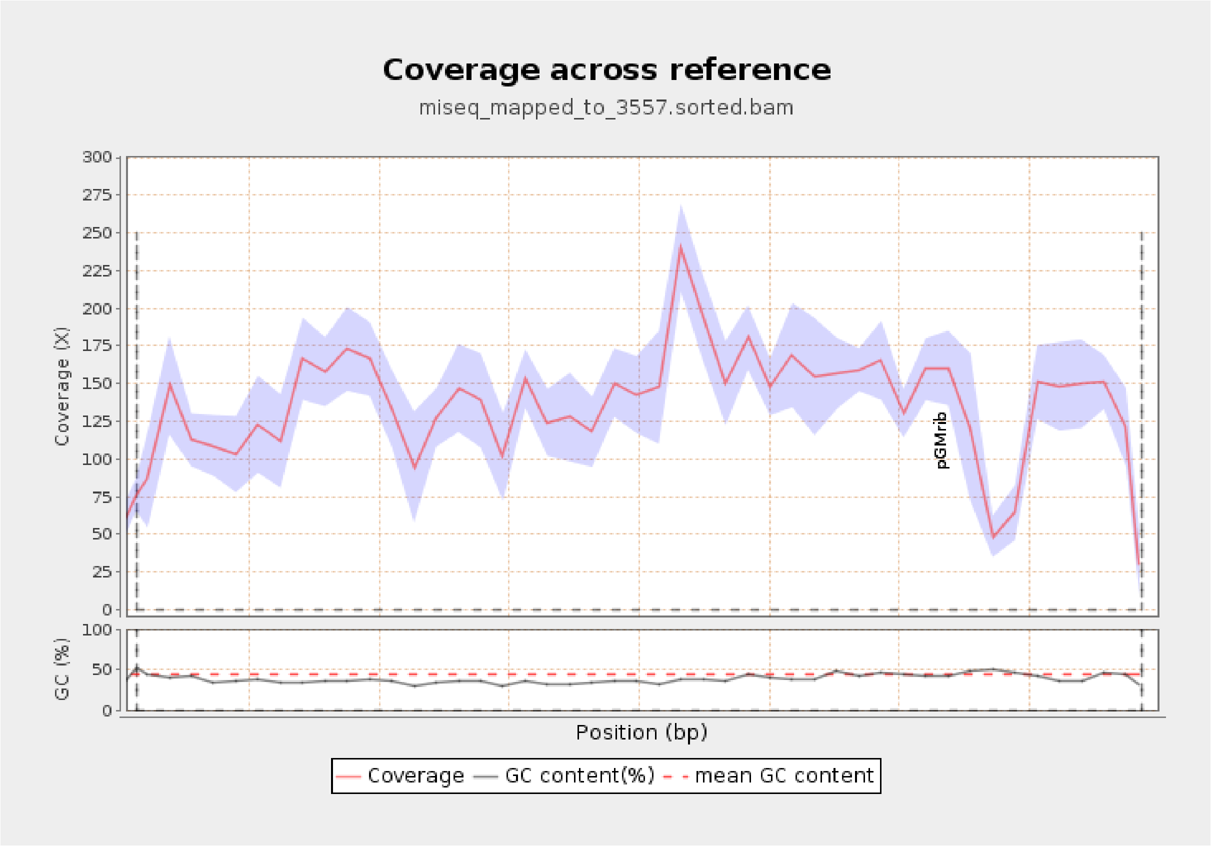
Coverage across reference
miseq_mapped_to_3557.sorted.bam
Coverage (X)
GC (%)
pGMrib
Position (bp)
Coverage
GC content(%)
mean GC content

C.

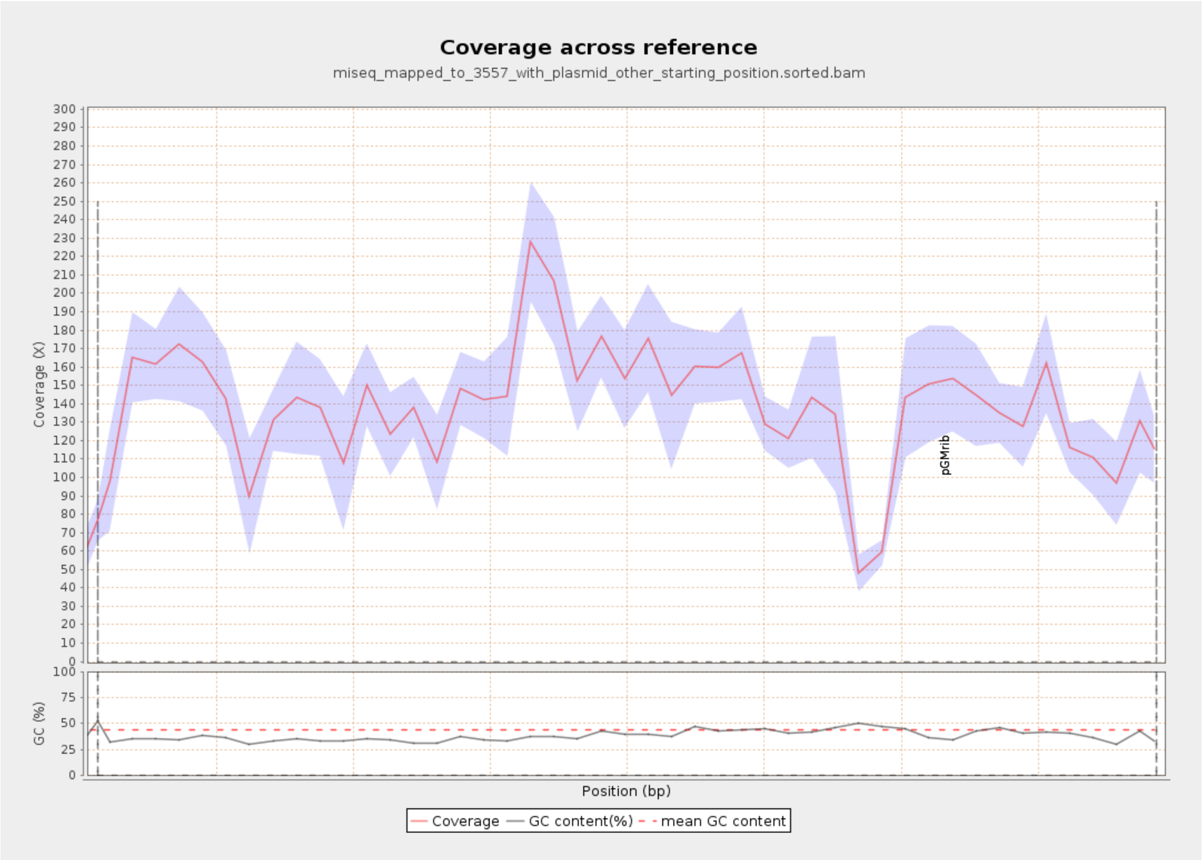
Coverage across reference
miseq_mapped_to_3557_with_plasmid_other_starting_position.sorted.bam
Coverage (X)
GC (%)
pGMrib
Position (bp)
Coverage
GC content(%)
mean GC content

D.

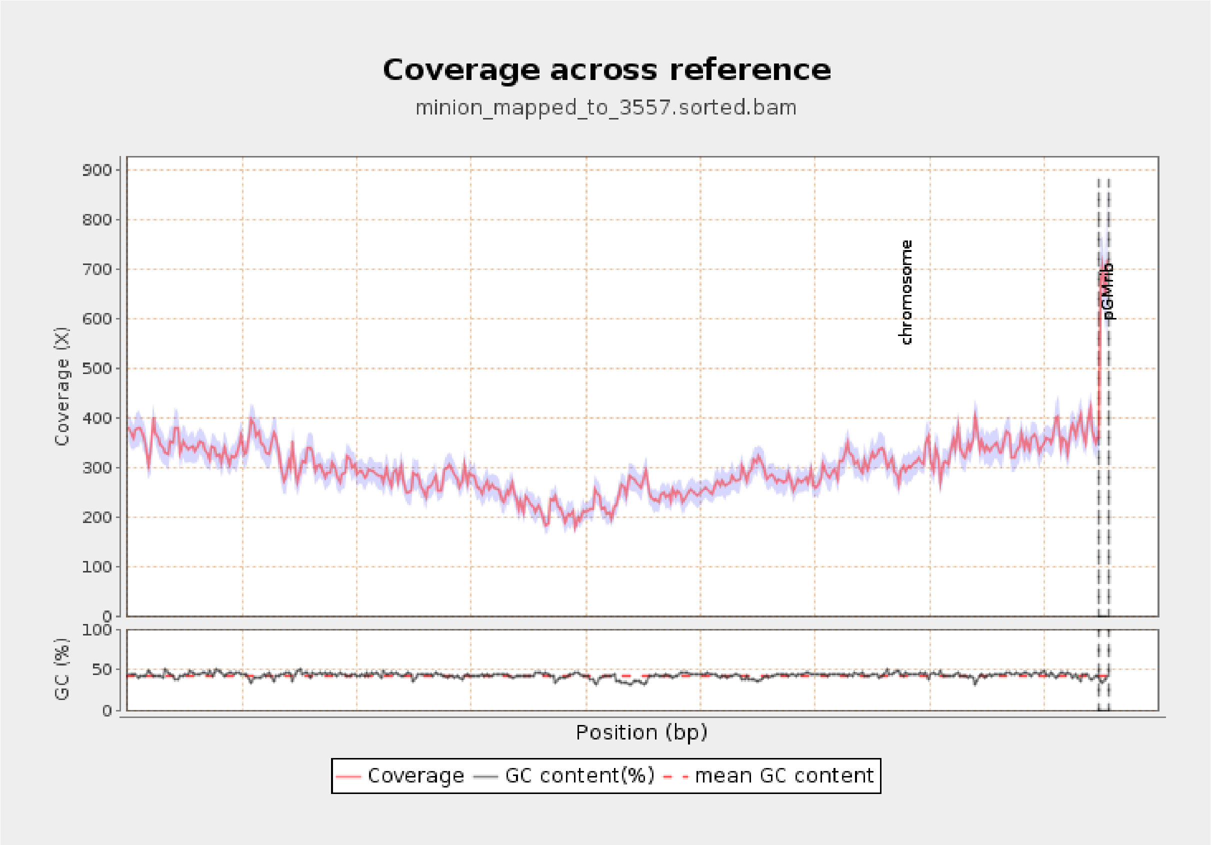

Coverage across reference
minion_mapped_to_3557.sorted.bam
Coverage (X)
900
800
700
600
500
400
300
200
100
0
chromosome
pGMrib
GC (%)
100
50
0
Position (bp)
Coverage
GC content(%)
mean GC content

E.

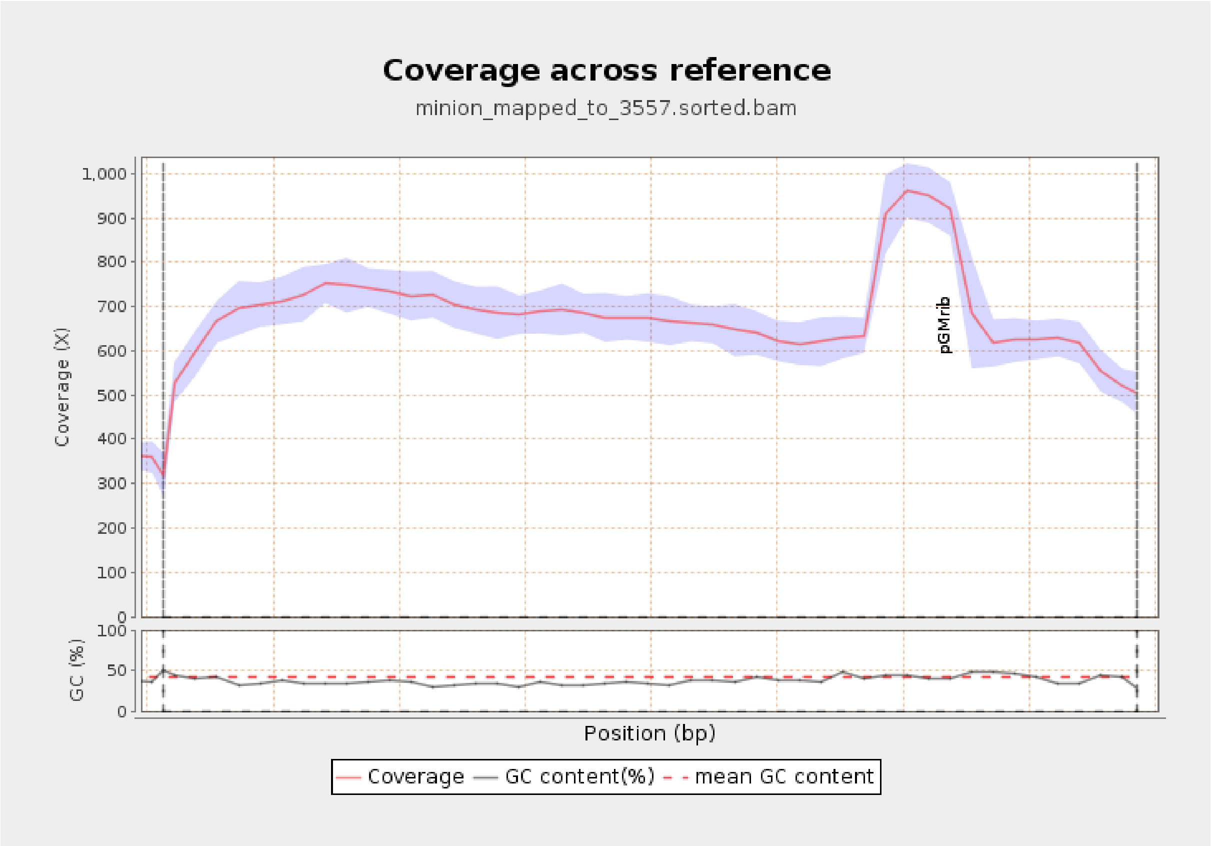

Coverage across reference
minion_mapped_to_3557.sorted.bam
Coverage (X)
1,000
900
800
700
600
500
400
300
200
100
0
pGMrib
GC (%)
100
50
0
Position (bp)
Coverage
GC content(%)
mean GC content

F.

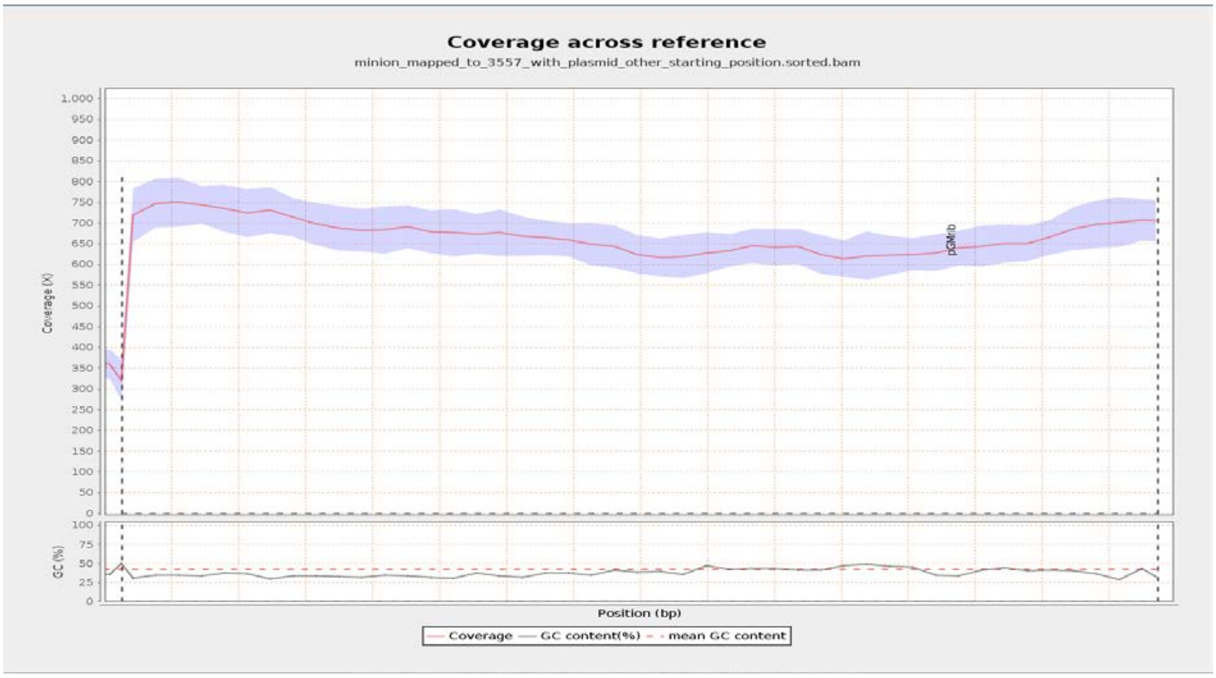
Coverage across reference
minion_mapped_to_3557_with_plasmid_other_starting_position.sorted.bam
Coverage (X)
GC (%)
Position (bp)
pGMrib
Coverage
GC content(%)
mean GC content

G.

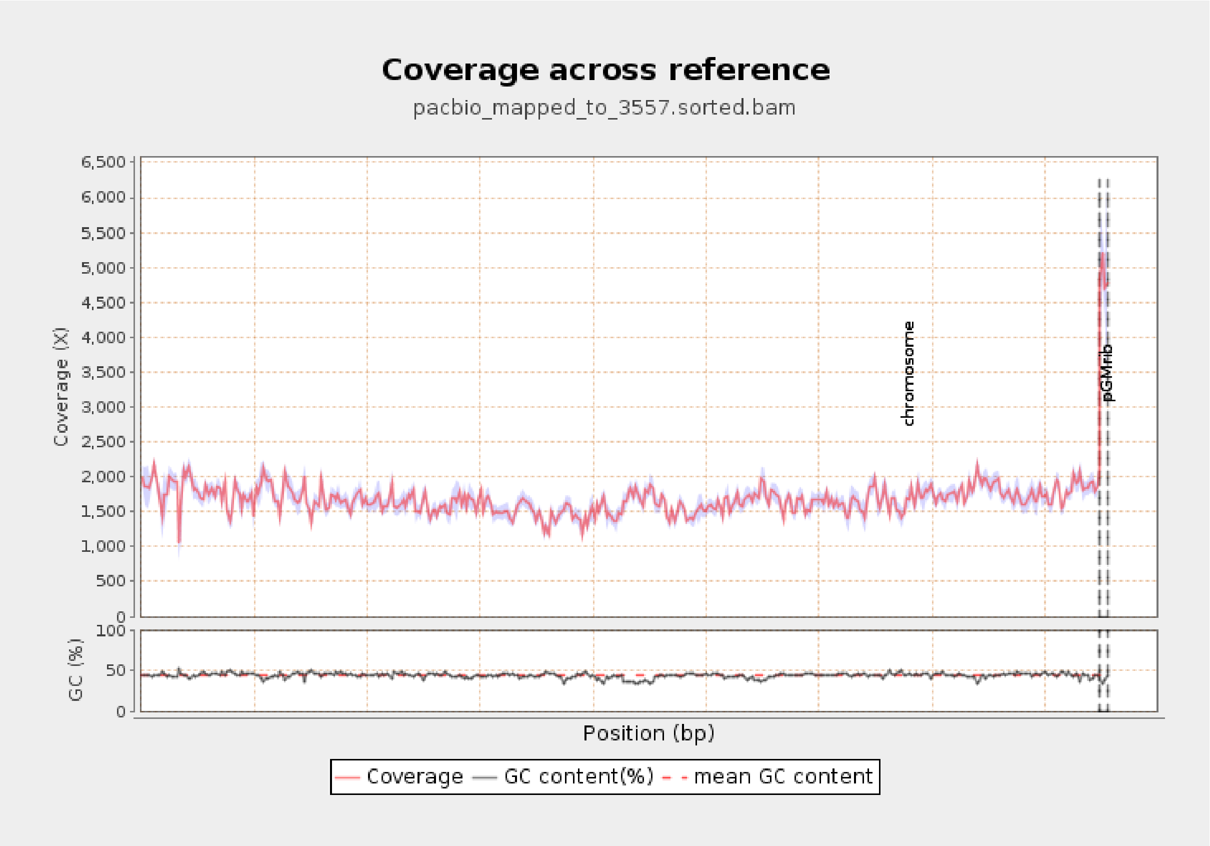
Coverage across reference
pacbio_mapped_to_3557.sorted.bam
Coverage (X)
GC (%)
chromosome
pGMrib
Position (bp)
Coverage
GC content(%)
mean GC content

H.

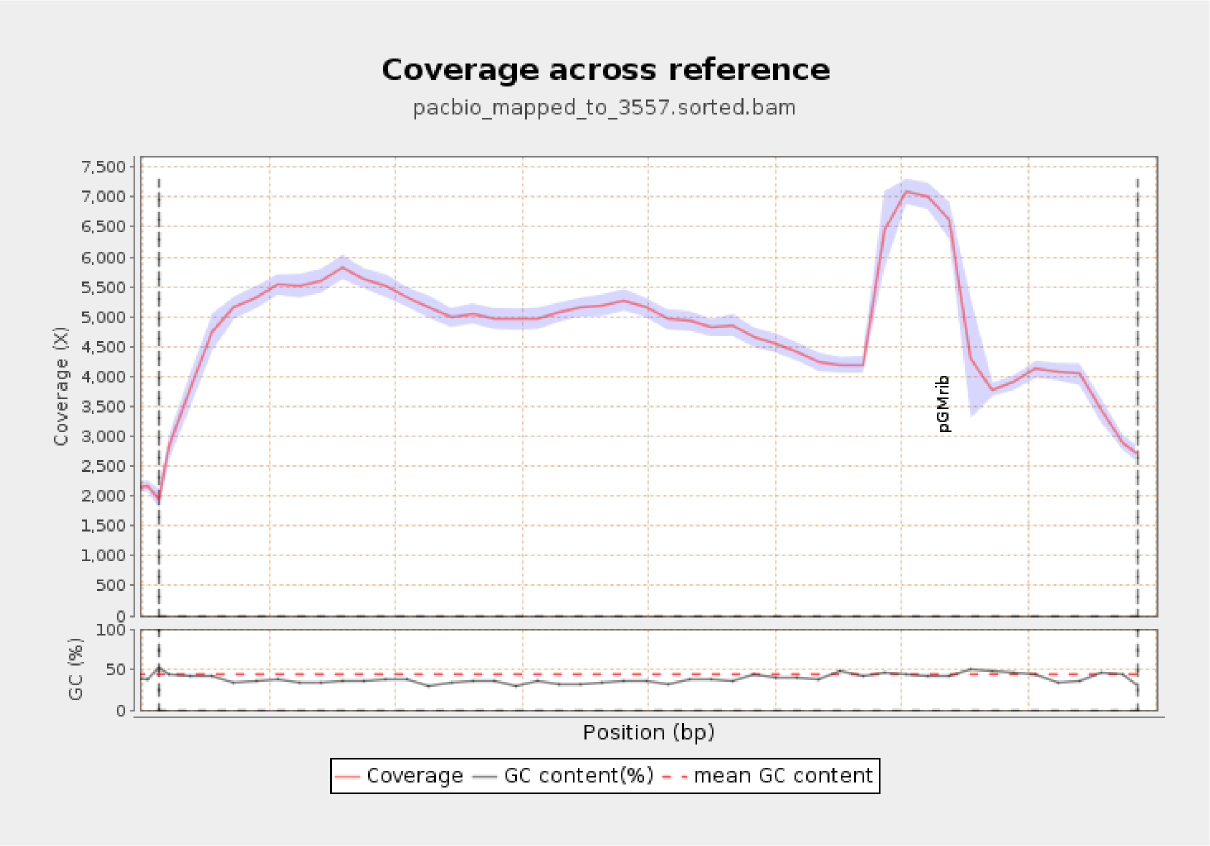

I.

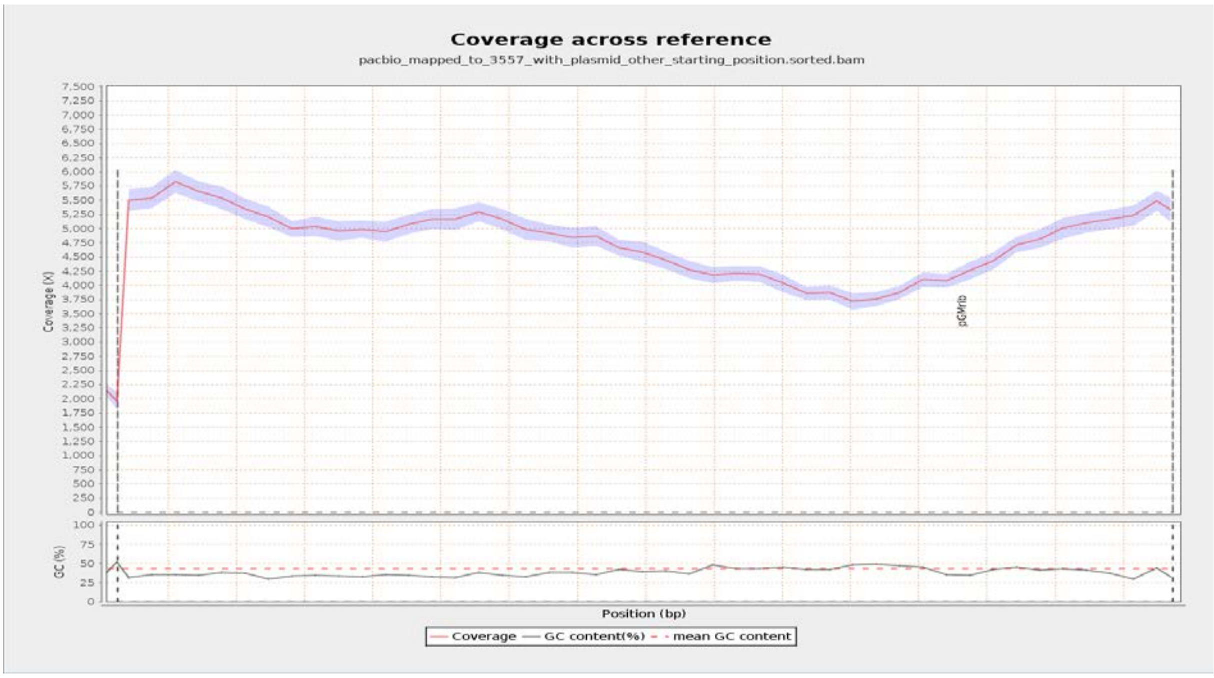

Figure S1: Average mapping coverage across each position in the *de novo* assembly of the GM *B. subtilis* 2014-3557 with MiSeq (A, B and C), MinION (D, E and F) and PacBio (G, H and I) reads visualized with Qualimap. Subfigures A, D and G visualise the coverage over both the chromosome and the plasmid (pGMrib), while subfigures B, D and F are zoomed in on pGMrib. Subfigures C, F and I are also zoomed in on pGMrib, but with another starting position (i.e. 5,000 bp further) to account for a repetitive region that caused a peak in coverage in subfigures E and H. With the red line the average coverage is shown while the blue area around the red line visualizes the standard deviation. Under each plot the GC% is shown. The dotted black line represents the separation of contigs, here it is where the plasmid contig starts and ends.

A.

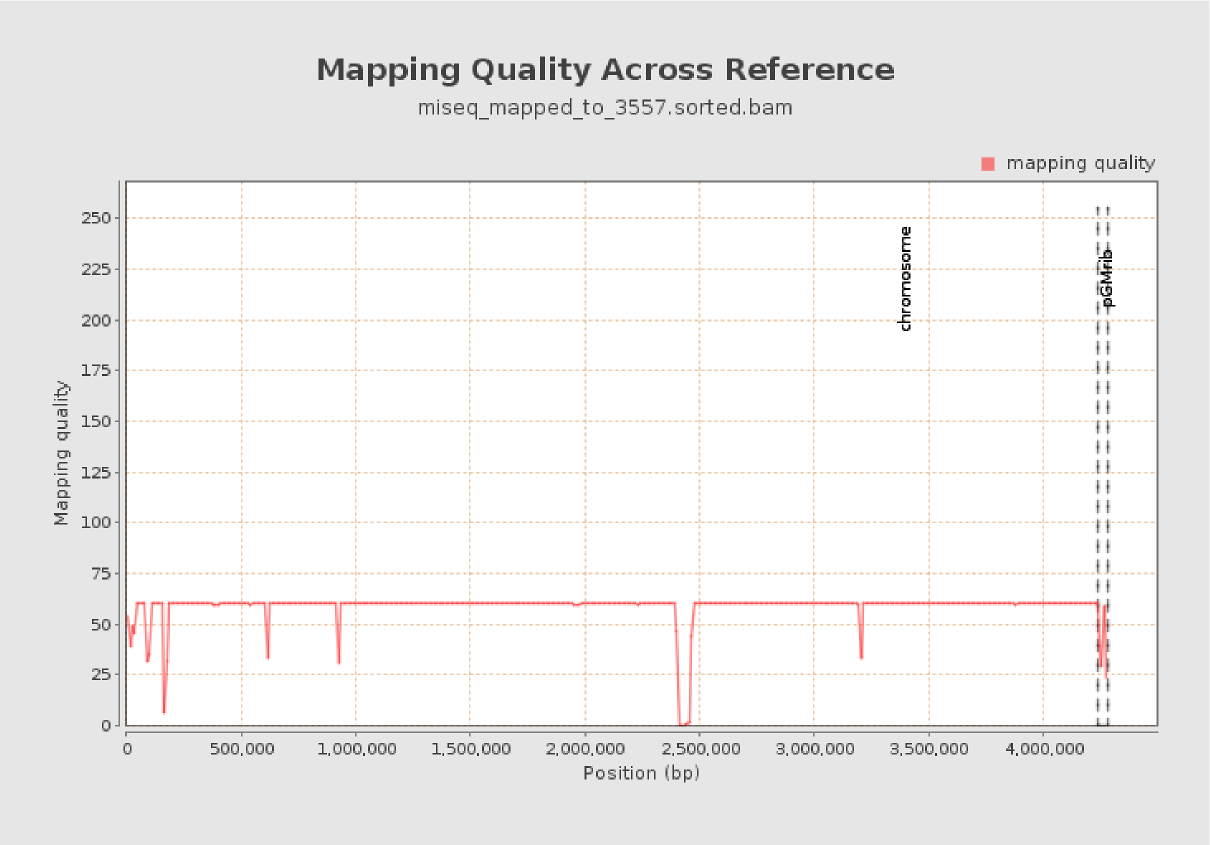
Mapping Quality Across Reference
miseq_mapped_to_3557.sorted.bam
mapping quality
Mapping quality
Position (bp)
chromosome
pGMrib
0
500,000
1,000,000
1,500,000
2,000,000
2,500,000
3,000,000
3,500,000
4,000,000
250
225
200
175
150
125
100
75
50
25

B.

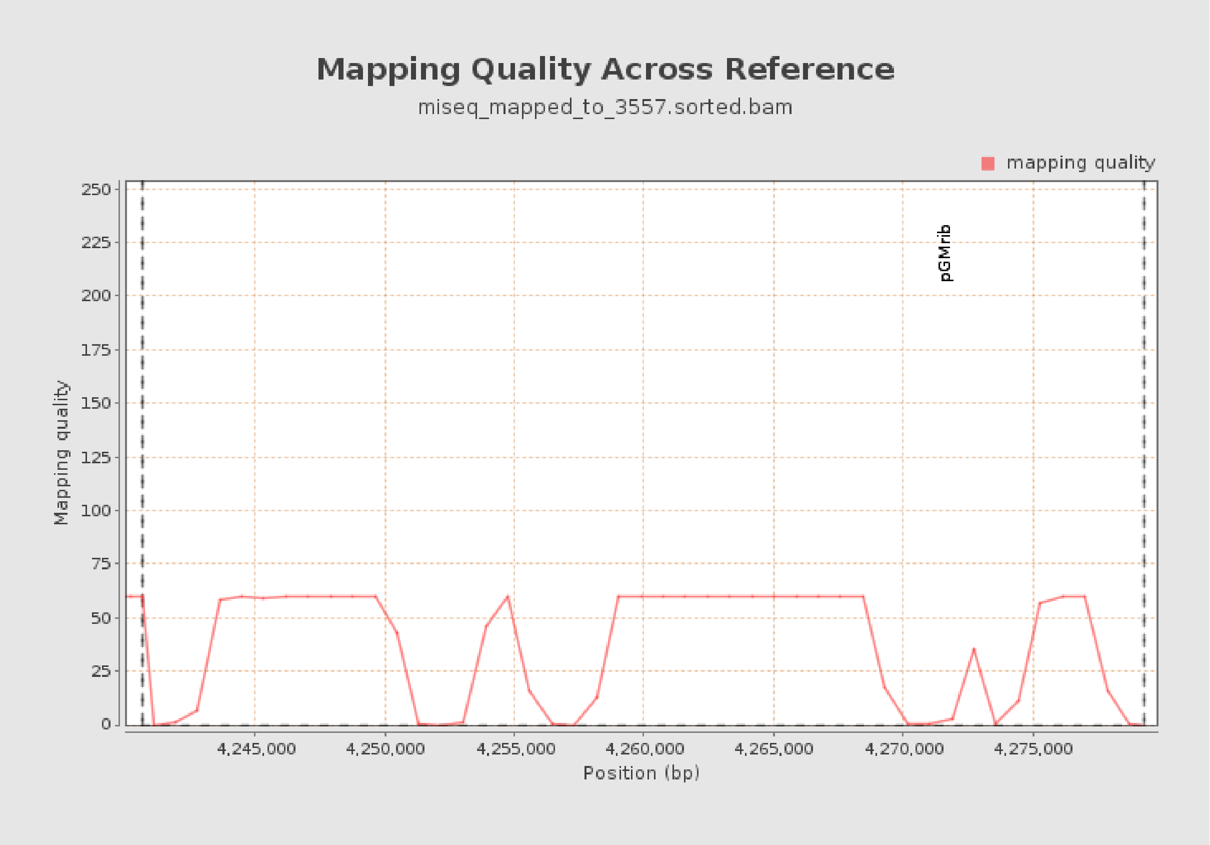
Mapping Quality Across Reference
miseq_mapped_to_3557.sorted.bam
mapping quality
Mapping quality
Position (bp)
pGMrib
4,245,000
4,250,000
4,255,000
4,260,000
4,265,000
4,270,000
4,275,000
250
225
200
175
150
125
100
75
50
25
0

C.

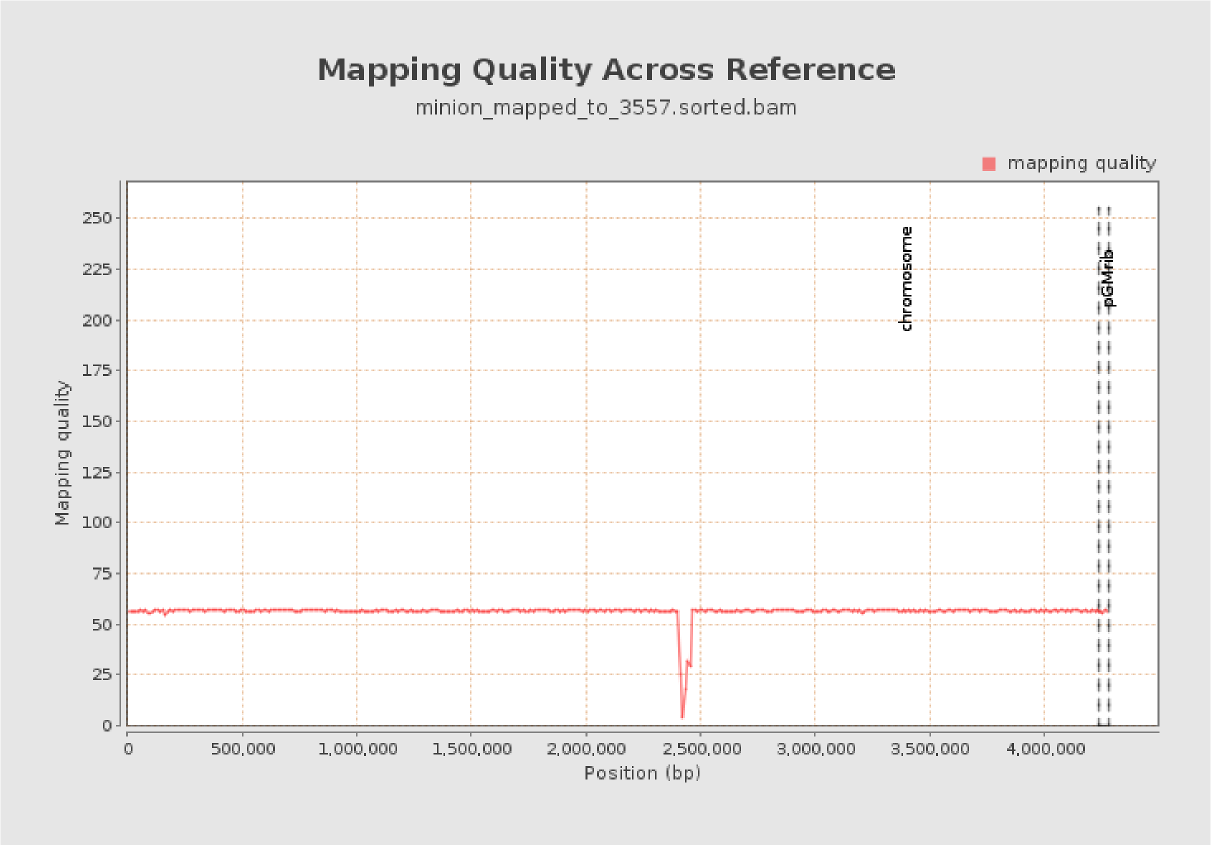
Mapping Quality Across Reference
minion_mapped_to_3557.sorted.bam
mapping quality
chromosome
pGMrib
Mapping quality
250
225
200
175
150
125
100
75
50
25
0
0
500,000
1,000,000
1,500,000
2,000,000
2,500,000
3,000,000
3,500,000
4,000,000
Position (bp)

D.

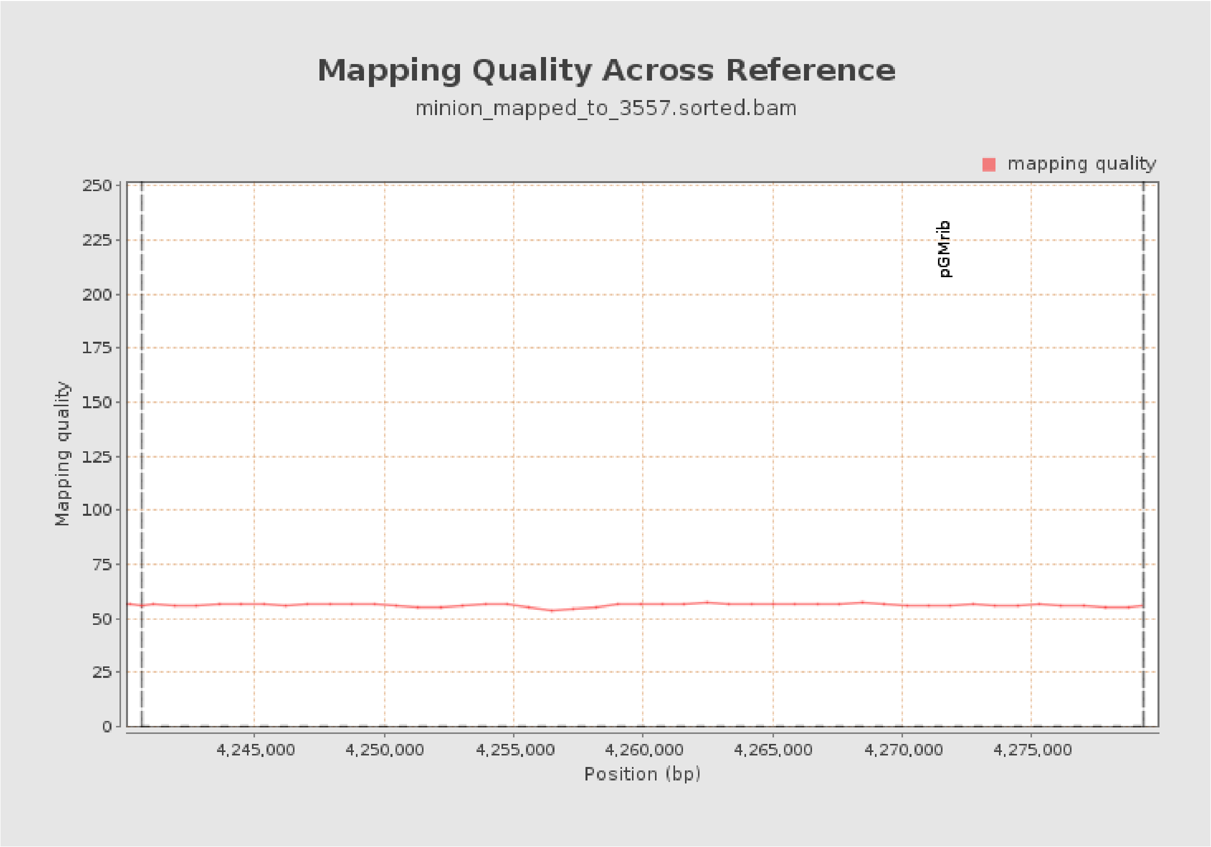
Mapping Quality Across Reference
minion_mapped_to_3557.sorted.bam
mapping quality
pGMrib
Mapping quality
250
225
200
175
150
125
100
75
50
25
0
4,245,000
4,250,000
4,255,000
4,260,000
4,265,000
4,270,000
4,275,000
Position (bp)

E.

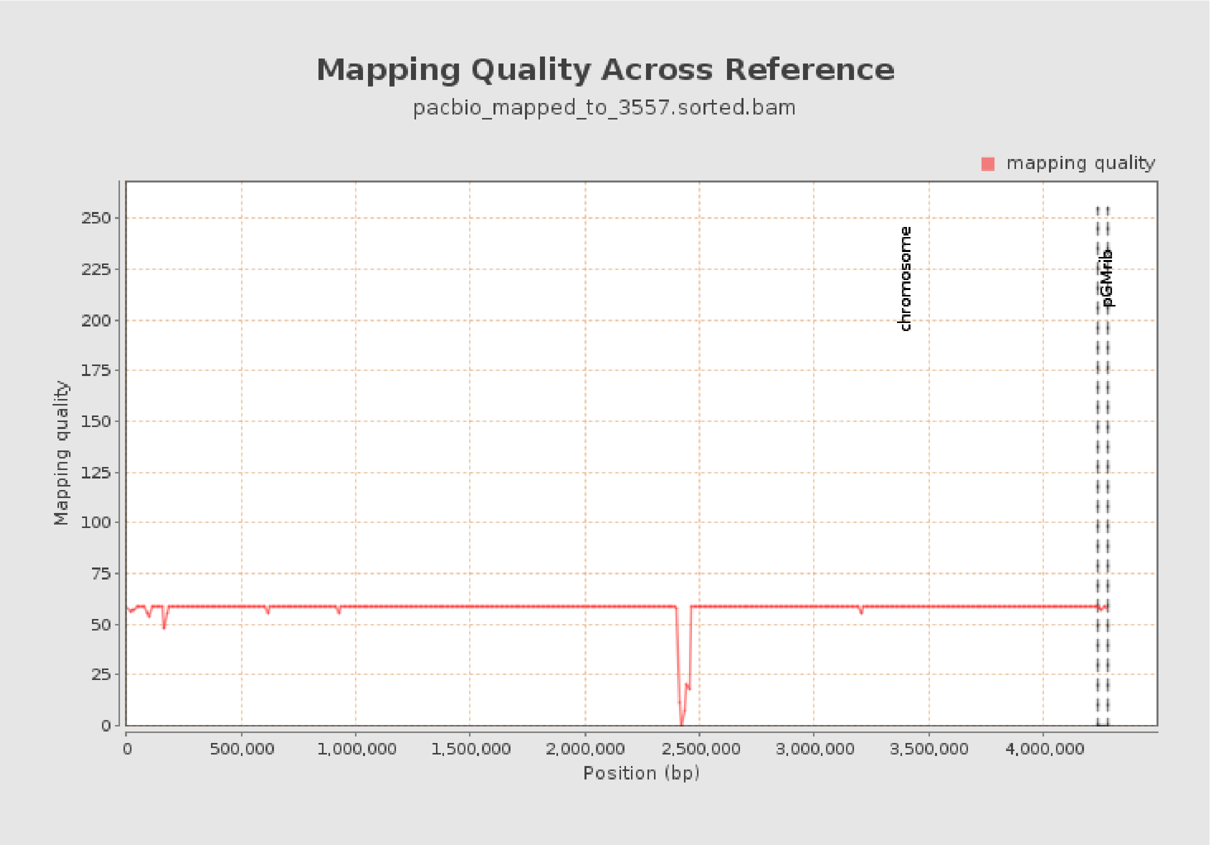

F.

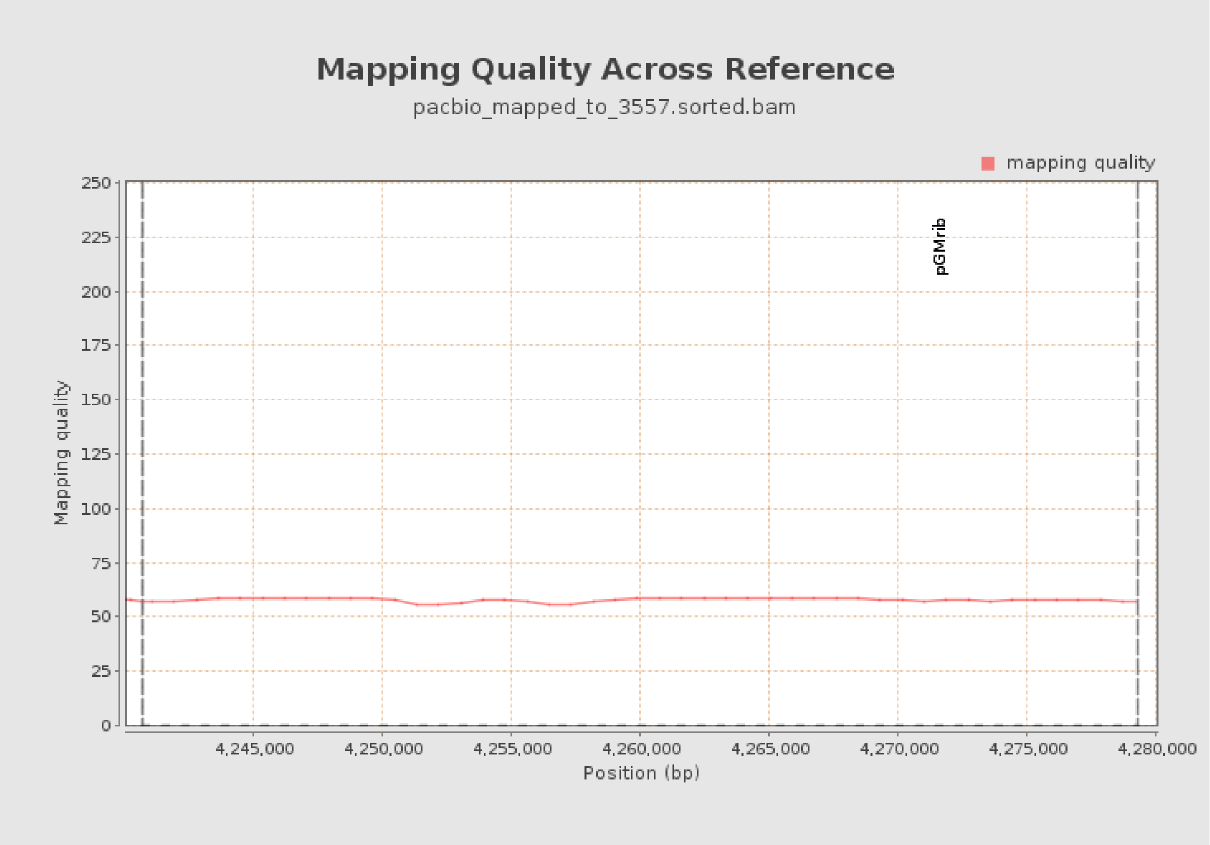

Figure S2: Average mapping quality across each position in the *de novo* assembly of the GM *B. subtilis* 2014-3557 with MiSeq (A and B), MinION (C and D) and PacBio (E and F) reads visualized with Qualimap. Subfigures A, C and E visualise the mapping quality over both the chromosome and the plasmid (pGMrib), while subfigures B, D and F are zoomed in on pGMrib. A mapping quality score of 60 means that the read

can only be uniquely mapped to one position, while a mapping quality score of 0 means that reads can map to multiple places in the genome. With only MiSeq reads (A), there are multiple regions in the chromosome (A) and the GM plasmid (B) where the quality mapping score of all reads is 0, which means that these reads can map on multiple places in the assembly, thereby impeding an accurate assembly. From position 2,406,920-2,459,995 a repetitive sequence (53kb region) with low mapping quality scores can be seen. However, the average mapping quality in the 53 kb repetitive region was higher for the MinION reads. With long reads (C-F)), the entire GM plasmid (D, F) is covered by uniquely mapped reads (Mapping score = 60). Reads from all three technologies could be uniquely mapped to the insertion sites of the 53 kb repetitive region. The dotted black line represents the separation of contigs, here it is where the plasmid contig starts and ends.

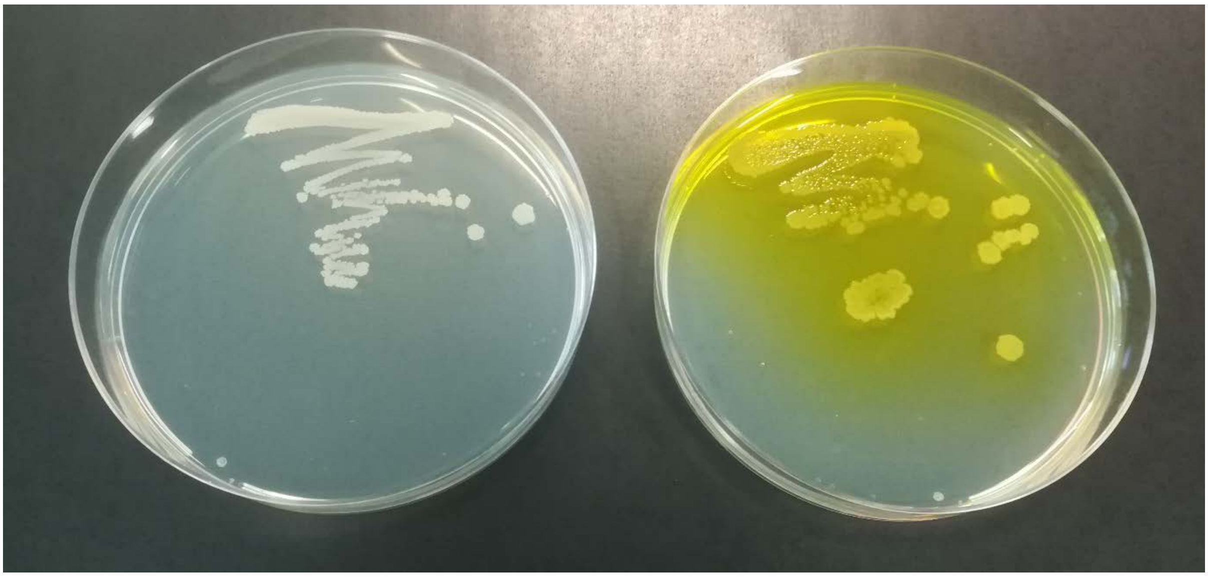

Figure S3: Culture plate of wild-type *B. subtilis* 168 (left) and GM *B. subtilis* 2014-3557 (right), after 48 hours of growth at 37 °C.
